# Quantum Thermodynamic Transformation Optics: A Unified Framework for Energy and Entropy With Application to the Casimir Force in Dissipative Metamaterials

**DOI:** 10.1002/nap2.70341

**Published:** 2026-11-02

**Authors:** Mohammad Mehdi Sadeghi, Mustafa Sarısaman

**Affiliations:** ^1^ Department of Physics Jahrom University Jahrom Iran; ^2^ Department of Physics Istanbul University Istanbul Türkey

**Keywords:** casimir effect, dissipative metamaterials, effective temperature, entropy production, quantum thermodynamic transformation optics (QTTO), quantum thermodynamics, thermofield dynamics, transformation optics

## Abstract

A novel concept, quantum thermodynamic transformation optics (QTTO), is introduced as a theoretical framework integrating transformation optics with thermodynamics of quantum dissipative systems. Beyond conventional coordinate transformations, QTTO describes how physically implemented transformation‐optical media with dispersion, loss, reservoir noise, and boundary conditions reorganize the statistical distribution of quantum energy and entropy‐related field fluctuations. Using thermofield dynamics, we establish transformation rules showing how local fluctuation related energy and entropy densities are controlled by the Jacobian determinant of the mapping. Geometric compression enhances local statistical weight of quantum and thermal fluctuations, whereas expansion produces the opposite effect, with global conservation laws and the second law of thermodynamics preserved. This framework provides a thermodynamic design rule for predicting quantum–thermal field correlations in dissipative metamaterials through coordinate‐induced optical geometry. As a practical test, we reformulate the Casimir effect, obtaining a reduced finite‐temperature pressure law that connects quantum vacuum and classical‐thermal regimes via a thermal weighting function. This relation shows how geometry and temperature jointly organize quantum pressure in an effective asymptotic description. Numerical results for Drude–Lorentz metamaterials support the analytical predictions and reproduce the relevant asymptotic behavior of the finite‐temperature Lifshitz–Matsubara formulation while capturing the overall crossover structure. To our knowledge, QTTO provides one of the first analytical foundations for a metric‐based thermodynamic framework of quantum fields in dissipative and curved optical media.

## Graphical abstract

Geometry in transformation optics can be extended to control quantum–thermal fluctuations, enabling redistribution of energy, entropy, and Casimir pressure in dissipative metamaterials.

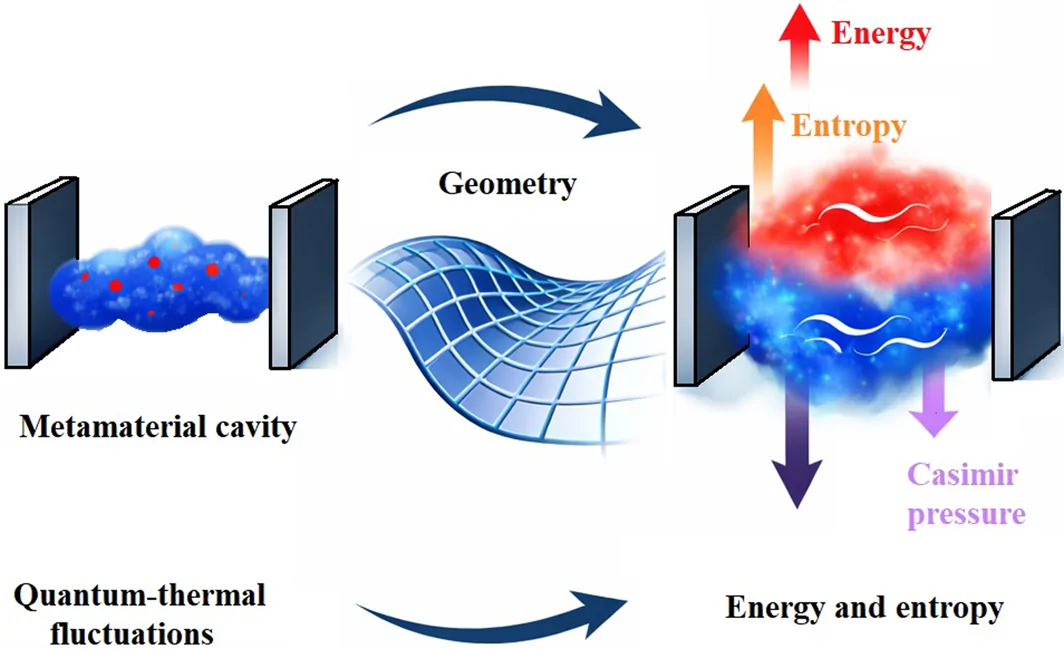

## 1 Introduction

Transformation optics (TO) provides a rigorous geometrical framework for manipulating electromagnetic propagation by mapping coordinate transformations onto spatially varying permittivity and permeability tensors [1, 2, 3, 4]. By embedding Maxwell's equations within an effective optical metric, TO has enabled the design of invisibility cloaks, field concentrators, and other metamaterial devices [5, 6, 7, 8]. However, conventional TO is primarily formulated as a classical field‐mapping theory. In its standard form, it does not explicitly address the quantum‐statistical character of electromagnetic fluctuations nor the thermodynamic consequences of dissipation, noise, and entropy production in realistic lossy media.

In dissipative metamaterials, electromagnetic fluctuations are constrained by the fluctuation–dissipation theorem, and the imaginary parts of the material response are inseparable from noise and heat generation [9]. This creates a conceptual gap: although TO prescribes how geometry reshapes fields, it does not by itself specify how geometry reorganizes quantum fluctuations, effective thermal occupation, local energy density, heat generation, and entropy‐related response. Bridging this gap requires a framework in which coordinate transformations, quantum statistics, and dissipative thermodynamics are treated together.

To address this problem, we introduce quantum thermodynamic transformation optics (QTTO) as a theoretical framework that combines transformation‐optical geometry with thermofield dynamics and reservoir‐based macroscopic quantum electrodynamics in dissipative media [10, 11, 12, 13, 14, 15, 16, 17]. The framework is intended for physically implemented transformation‐optical media, where a coordinate mapping is realized through spatially structured material tensors with dispersion, loss, reservoir noise, and boundary conditions. Thus, QTTO should not be understood as assigning thermodynamic effects to a passive coordinate transformation alone. Rather, it provides a metric‐based description of how an implemented transformation medium reorganizes finite‐temperature electromagnetic fluctuations in a manner consistent with the fluctuation–dissipation theorem.

A central consequence of QTTO is the emergence of a metric‐dependent effective statistical temperature,

$$T_{\text{eff}}\left(r\right)=\frac{T}{\left|\det\;\Lambda \left(r\right)\right|}$$

which enters the transformed field correlations and the metric‐dependent fluctuation–dissipation relation. This quantity does not represent a literal change of the external bath temperature, nor does it imply physical heating or cooling generated by geometry alone. Instead, $T_{\text{eff}}$ is introduced as an operational parameter that summarizes how the implemented optical metric modifies the local statistical weight of quantum and thermal fluctuations under the assumptions of local equilibrium, passivity, and slowly varying material profiles.

Within thiscontrolled interpretation, we derive transformation rules for fluctuation‐related energy density, heat generation, and entropy‐related response. These laws describe how local fluctuation observables are redistributed by the implemented optical geometry, whereas the corresponding global balance laws remain preserved. In this sense, QTTO extends transformation optics from a purely classical field‐mapping scheme to an effective thermodynamic organization of macroscopic‐QED correlations in dissipative transformation media, without implying the generation of thermodynamic resources by a coordinate transformation alone.

As a concrete application, we reformulate the finite‐temperature Casimir pressure in a dissipative transformed cavity [18, 19, 20, 21, 22]. The revised QTTO pressure law separates the response into a vacuum‐dominated branch and a thermally dominated branch. These branches are connected by a smooth weighting function

$$W\left(\tau \right)=1-\exp\left(-\tau^{\nu }\right),\tau =\frac{T_{\text{eff}}}{T_{⋆}}$$

where $T_{⋆}$ is a cavity reference crossover scale rather than a second effective temperature. The resulting expression should be understood as a reduced asymptotic interpolation rather than a first‐principles replacement for the full Lifshitz–Matsubara theory. The full Lifshitz formulation retains the complete spectral information, whereas the QTTO pressure law isolates the reduced thermodynamic organization of the response into geometrically controlled quantum and thermal branches [23, 24, 25, 26, 27, 28, 29, 30].

The revised numerical comparison with the finite‐temperature Lifshitz–Matsubara theory shows that the QTTO pressure law reproduces the correct low‐temperature quantum limit, captures the high‐temperature thermal asymptote, and describes the overall crossover structure for a Drude–Lorentz dissipative cavity [18, 19, 20, 21]. The crossover parameters are determined by the spectral properties of the cavity and are calibrated against the corresponding macroscopic‐QED/Lifshitz calculation. This clarification also specifies the range of validity of the reduced QTTO pressure law and distinguishes it from an exact point‐by‐point evaluation of the Lifshitz–Matsubara sum.

The paper is organized as follows. Section 2 formulates thermofield quantization in a curved optical metric and explains how dissipation is included through a reservoir‐based macroscopic‐QED construction. Section 3 derives transformation laws for energy density, heat generation, and entropy‐related response. Section 4 applies the framework to finite‐temperature Casimir pressure in dissipative metamaterials. Section 5 summarizes the implications and future directions. Detailed derivations and numerical validations are provided in Supporting Information S1: Appendices A–D.

## 2 Thermofield Quantization in a Curved Optical Metric (Complete)

The QTTO formalism is built upon the finite‐temperature thermofield dynamics (TFD) formalism originally developed by Takahashi and Umezawa [16, 17] and later applied to quantum optics and dissipative media [13, 14]. QTTO extends TFD by embedding the doubled Hilbert space within a curved optical metric generated by a transformation‐optics mapping. This embedding transforms the theory into a covariant statistical field theory in which energy, heat, and entropy transform together with the metric tensor. Below, we (i) recall the essentials of TFD, (ii) introduce the optical metric arising from coordinate transformations, (iii) formulate the curved‐metric thermofield Lagrangian for dissipative systems, and (iv) derive the Hamiltonian, equal‐time commutators, Green tensor, and the fluctuation–dissipation theorem (FDT) in the effective metric.

### 2.1 Thermofield Dynamics: Doubling, Thermal Vacuum, and Operators

In TFD, the total Hilbert space is doubled,

$$H_{T}=H\;⊗\;\tilde{H}$$

and for each physical mode $a_{\lambda },a_{\lambda }^{†}$ one introduces a thermal partner ${\tilde{a}}_{\lambda },{\tilde{a}}_{\lambda }^{†}$. Thermal averages are pure‐state expectations in the thermal vacuum ∣0(β)〉 with $\beta ={\left(k_{B}T\right)}^{-1}$:

$${\langle O\rangle }_{T}=\langle 0\left(\beta \right)\;|\;O\;|\;0\left(\beta \right)\rangle ,{\langle a_{\lambda }^{†}a_{\lambda^{\prime }}\rangle }_{T}=\delta_{\lambda \lambda^{\prime }}\;n_{T}\left(\omega_{\lambda }\right),n_{T}\left(\omega \right)=\frac{1}{e^{\beta \hbar \omega }-1}$$

This formalism preserves canonical commutation relations at finite temperature while maintaining thermodynamic consistency [11, 12, 13].

We employ the Lorenz gauge for the four‐potential $A^{\mu }$, and represent dissipation and dispersion using a Huttner–Barnett‐type reservoir model, which provides a consistent Hamiltonian description of lossy and dispersive media in the TFD framework [8, 9, 10].

### 2.2 Optical Metric From a Coordinate Mapping

A smooth coordinate mapping $x^{\prime i}=f^{i}\left(x^{j}\right)$ with Jacobian $\Lambda_{j}^{i}=\partial x^{\prime i}/\partial x^{j}$ induces the optical metric

$$g_{ij}={\left(\Lambda^{-1}\Lambda^{-T}\right)}_{ij},|g|=\det\;g_{ij}={\left|\det\;\Lambda \right|}^{-2}.$$

In this picture, Maxwell's equations retain their form, whereas effective material tensors transform as follows [1, 2, 3, 4]:

$$\varepsilon_{ij}^{\prime }=\frac{1}{\left|\det\;\Lambda \right|}\;\Lambda_{\;i}^{m}\Lambda_{\;j}^{n}\;\varepsilon_{mn},\mu_{ij}^{\prime }=\frac{1}{\left|\det\;\Lambda \right|}\;\Lambda_{\;i}^{m}\Lambda_{\;j}^{n}\;\mu_{mn},$$

and spatial derivatives are replaced by covariant derivatives compatible with $g_{ij}$.

### 2.3 Coordinate Covariance Versus Physical Metamaterial Implementation

A distinction must be made between a passive coordinate transformation and a physical transformation‐optical implementation. A coordinate transformation by itself is a change of description and cannot generate heat, entropy production, local cooling, or a change of thermodynamic temperature. In conventional transformation optics, the physical content appears only when the mapping is implemented by a material distribution whose constitutive tensors reproduce the transformed Maxwell equations.

The same distinction is essential in QTTO. The thermodynamic quantities discussed below are not assigned to geometry as an abstract coordinate change. They refer to a physically implemented transformation medium characterized by spatially structured, frequency‐dependent, and generally lossy tensors $\varepsilon^{\prime ij}\left(r,\omega \right)$ and $\mu^{\prime ij}\left(r,\omega \right)$, together with the reservoir degrees of freedom and noise correlations required by macroscopic quantum electrodynamics. Thus, the optical metric enters the theory through the material implementation, its dispersion, its absorption channels, and its boundary conditions.

Within this interpretation, the metric‐dependent effective temperature introduced below should be understood as an operational parameter in the transformed fluctuation–dissipation relation. It does not represent a literal thermodynamic temperature field imposed on the medium. Rather, it parametrizes the change in the local statistical weight of field fluctuations when the physical metamaterial implementation modifies the mode normalization, local density of states, and Green tensor. Therefore, statements about geometric amplification or suppression of energy‐ and entropy‐related quantities should be read as statements about fluctuation observables in the implemented dissipative medium, not as claims that a coordinate transformation alone produces thermodynamic resources.

This clarification also fixes the scope of the following derivation. The transformation laws derived in Section 3 are obtained under the assumptions of passivity, local equilibrium, causal dispersion, and a slowly varying transformation medium, so that a local macroscopic‐QED description and a local fluctuation–dissipation relation remain meaningful. Outside these assumptions, the full nonlocal and nonequilibrium response of the material would have to be treated explicitly.

### 2.4 Curved‐Metric Thermofield Lagrangian With Dissipation

To incorporate both dissipation and curvature within a consistent quantum framework, we generalize the standard thermofield reservoir model to an optical metric generated by a transformation‐optics mapping. This approach builds upon the Huttner–Barnett representation of absorptive media [8, 9] and its thermofield extension by Amooghorban et al. [10, 13], but introduces two essential innovations:

1. A metric weighting of all field and reservoir terms through the geometric factor √|g|, and
2. An effective statistical embedding that relates the implemented optical metric to the local weighting of finite‐temperature field correlations.

This expression represents the metric–thermodynamic generalization of the standard thermofield Lagrangian and forms the foundational basis of the QTTO formalism.”

(1) $$\begin{array}{cc} L_{\text{QTTO}}=\; & \frac{\sqrt{|g|}}{2}\left(E_{i}^{\prime }\;\varepsilon^{\prime \;ij}\left(r,\omega ;T\right)\;E_{j}^{\prime }-B_{i}^{\prime }\;\mu^{\prime \;ij}\left(r,\omega ;T\right)\;B_{j}^{\prime }\right)\\ & \;-\sqrt{|g|}\int_{0}^{\infty }d\omega \;\left[{X_{\omega }^{\prime }}^{\;†}\left(\partial_{t}^{2}+\omega^{2}\right)X_{\omega }^{\prime }+{Y_{\omega }^{\prime }}^{\;†}\left(\partial_{t}^{2}+\omega^{2}\right)Y_{\omega }^{\prime }\right]\\ & \;+L_{\mathrm{int}}\;\left[A_{\mu }^{\prime };X_{\omega }^{\prime },Y_{\omega }^{\prime };T\right]+{\tilde{L}}_{\text{QTTO}}, \end{array}$$

where $\text{Im}\;\varepsilon^{\prime }>0$ and $\text{Im}\;\mu^{\prime }>0$ enforce causality and passivity, consistent with the Kramers–Kronig relations [15, 16]. The primed quantities denote fields defined in the transformed metric $g_{ij}$, related to the coordinate mapping $x^{\prime i}=f^{i}\left(x^{j}\right)$ introduced in Section 2.2.

The electric and magnetic fields $E_{i}^{\prime }$ and $B_{i}^{\prime }$ are constructed from the four‐potential $A_{\mu }^{\prime }$ using covariant derivatives compatible with $g_{ij}$. The additional term ${\tilde{L}}_{\text{QTTO}}$ represents the “tilde” thermal sector that preserves canonical symmetry in the doubled Hilbert space, a property unique to the QTTO construction.

Dissipation is included in the transformation‐optics formalism by transforming the full dispersive response, not only the lossless constitutive tensors. Thus, after the coordinate mapping, the tensors $\varepsilon^{\prime \text{ij}}\left(r,\omega \right)$ and $\mu^{\prime \text{ij}}\left(r,\omega \right)$ remain complex and frequency‐dependent, with $\text{Im}\varepsilon^{\prime }>0$ and $\text{Im}\mu^{\prime }>0$ enforcing passivity and causality. The reservoir fields in Equation (1) represent the microscopic degrees of freedom responsible for absorption and are embedded in the same optical metric as the electromagnetic field—note the metric factor $\sqrt{\left|g\right|}$ multiplying the reservoir kinetic terms. Consequently, the transformation acts simultaneously on the electromagnetic field, the material response, and the reservoir sector. The corresponding noise correlations are then fixed by the metric‐dependent fluctuation‐dissipation theorem (Equation (9)), rather than being added phenomenologically. The tilde sector ${\tilde{L}}_{\text{QTTO}}$ ensures that the canonical structure of thermofield dynamics is preserved in the curved metric, allowing a consistent treatment of finite‐temperature dissipation.

By embedding both the field and its thermal conjugate within a curved optical metric, this formulation extends thermofield dynamics from a statistical description of dissipation to a covariant theory of energy, work, and entropy. This geometric embedding constitutes one of the core theoretical innovations of the QTTO framework, clearly distinguishing it from earlier thermofield and macroscopic QED formulations.

### 2.5 Canonical Momenta and Equal‐Time Commutators

The canonical momentum conjugate to $A_{i}^{\prime }$ (Lorenz gauge) is

(2) $$\Pi^{\prime i}\left(r,t\right)=\frac{\partial L_{\text{QTTO}}}{\partial \left(\partial_{t}A_{i}^{\prime }\right)}=\sqrt{\left|g\right|}\;\varepsilon^{\prime ij}\;E_{j}^{\prime }.$$

Equal‐time commutators take the metric‐weighted form

(3) $$\left[A^{\prime i}\left(r,t\right),\Pi^{\prime j}\left(r^{\prime },t\right)\right]=i\hbar \;\delta^{ij}\;\delta^{\left(3\right)}\;\left(r-r^{\prime }\right)\;\sqrt{\left|g\right|}$$

Moreover, for the reservoirs,

$$\left[X_{\omega }^{\prime }\left(r\right),{X_{\omega^{\prime }}^{\prime }}^{\;†}\left(r^{\prime }\right)\right]=\delta \left(\omega -\omega^{\prime }\right)\delta^{\left(3\right)}\left(r-r^{\prime }\right)\;\sqrt{\left|g\right|}$$

The factor $\sqrt{|g|}$ guarantees energy normalization under the mapping.

### 2.6 Hamiltonian Density in the Optical Metric

A Legendre transform of the metric‐weighted thermofield Lagrangian gives the Hamiltonian density of the transformed dissipative medium as

(4) $$H_{T}^{\prime }=\frac{\sqrt{\left|g\right|}}{2}\left(E_{i}^{\prime }\;\varepsilon^{\prime ij}\;E_{j}^{\prime }+B_{i}^{\prime }\;\mu^{\prime ij}\;B_{j}^{\prime }\right)+H_{\text{res}}^{\prime }\left(X_{\omega }^{\prime },Y_{\omega }^{\prime }\right)+{\tilde{H}}_{T}^{\prime }$$

Here, the first term represents the electromagnetic contribution in the implemented optical metric, $H_{\text{res}}^{\prime }$ denotes the reservoir Hamiltonian responsible for absorption and dispersion, and ${\tilde{H}}_{T}^{\prime }$ is the tilde‐sector Hamiltonian required by thermofield dynamics. The factor $\sqrt{|g|}$ ensures the covariant normalization of field and reservoir energies in the transformed medium.

The thermal expectation value of the Hamiltonian density may be written in terms of the transformed Green tensor as

(5) $$\langle H_{T}^{\prime }\rangle =\frac{\hbar }{\pi }\int_{0}^{\infty }d\omega \omega \;\coth\left(\frac{\hbar \omega }{2k_{B}T_{\text{eff}}\left(r\right)}\right)\text{ImTr}\left[\varepsilon^{\prime }\left(r,\omega \right)G^{\prime }\left(r,r;\omega \right)\right],$$

where $G^{\prime }$ is the electric Green tensor in the optical metric (Section 2.7). The temperature entering Equation (5) is not introduced as a new thermodynamic bath temperature. It is obtained by rewriting the transformed local fluctuation spectrum in the standard Bose/FDT form after the coordinate mapping has been implemented by a dispersive and dissipative medium.

In the original physical medium, the reservoir is assumed to be in local thermal equilibrium at the bath temperature T. Under a transformation‐optical implementation, the coordinate mapping modifies the covariant volume element, the normalization of electromagnetic modes, and the local Green tensor. For a slowly varying passive medium, these changes can be collected into a local Jacobian factor in the fluctuation spectrum. Equivalently, the transformed correlation function may be written in the same functional form as the standard equilibrium fluctuation–dissipation relation, but with an effective statistical parameter multiplying the Bose occupation weight. Matching the transformed occupation weight to the standard form gives

(6) $$T_{\text{eff}}\left(r\right)\equiv \frac{T}{\left|\det\;\Lambda \left(r\right)\right|}$$

Thus, $T_{\text{eff}}$ is an operational effective statistical temperature defined by the local representation of the transformed field correlations. It is not an independently imposed temperature field and does not imply that the material has been physically heated or cooled. The physical bath temperature remains T. The role of $T_{\text{eff}}$ is only to parametrize how the implemented optical metric changes the local statistical weight of quantum and thermal fluctuations through mode normalization, density‐of‐states redistribution, and the metric‐dependent Green tensor.

Consequently, in the local effective representation, a compressive implemented mapping (∣detΛ∣<1) assigns a larger statistical weight to the thermal part of the transformed fluctuation spectrum, whereas an expansive mapping assigns a smaller weight. This should not be interpreted as a physical increase or decrease of the material temperature. The bath temperature remains fixed at T, whereas $T_{\text{eff}}$ only parametrizes the Jacobian‐dependent redistribution of quantum and thermal field correlations in the implemented dissipative medium.

This construction is local and approximate in the same sense as the use of local constitutive tensors in transformation optics and macroscopic QED. It assumes passivity, causal dispersion, local thermal equilibrium of the reservoir, and sufficiently smooth spatial variation of the transformation medium. Under these assumptions, $T_{\text{eff}}$ provides a compact way to express the transformed fluctuation spectrum while preserving the standard equilibrium structure of the fluctuation–dissipation theorem.

### 2.7 Mode Expansion and Normalization in $g_{ij}$

A covariant mode expansion is

(7) $$E^{\prime }\left(r,t\right)=\sum_{\lambda }\;\sqrt{\frac{\hbar \omega_{\lambda }}{2}}\;\left[u_{\lambda }\left(r\right)\;a_{\lambda }\left(t\right)+h.c.\right],\int d^{3}x\;\sqrt{\left|g\right|}\;\varepsilon^{\prime \;ij}\;u_{\lambda ,i}^{*}u_{\lambda^{\prime },j}=\delta_{\lambda \lambda^{\prime }}.$$

The $\sqrt{|g|}$ weight preserves total modal energy under the mapping. The eigenfrequencies $\omega_{\lambda }$ depend on $g_{ij}$ via the effective Maxwell operator.

### 2.8 Green Tensor and Fluctuation–Dissipation Relation (FDT)

The Green tensor $G_{ij}^{\prime }$ satisfies (in frequency domain)

(8) $$\nabla^{\prime }\times \mu^{\prime -1}\left(r,\omega \right)\;\nabla^{\prime }\times G^{\prime }\left(r,r^{\prime };\omega \right)-\omega^{2}\varepsilon^{\prime }\left(r,\omega \right)\;G^{\prime }\left(r,r^{\prime };\omega \right)=I\;\frac{\delta^{\left(3\right)}\left(r-r^{\prime }\right)}{\sqrt{\left|g\right|}}.$$

The metric‐FDT reads

(9) $${\langle E^{\prime i}\left(r\right)\;E^{\prime j}\left(r^{\prime }\right)\rangle }_{T}=\frac{\hbar }{\pi }\int_{0}^{\infty }d\omega \omega \;\coth\left(\frac{\hbar \omega }{2k_{B}T_{\text{eff}}\left(r\right)}\right)\text{Im}\;G^{\prime ij}\left(r,r^{\prime };\omega \right)$$

Equation (9) should be read as the local effective FDT representation of the transformed dissipative medium under the assumptions stated above. The physical reservoir remains at the bath temperature T, whereas $T_{\text{eff}}$ parametrizes the Jacobian‐induced redistribution of the fluctuation spectrum in the implemented optical metric. Therefore, this relation is not a new equilibrium law for a coordinate transformation alone, but a compact macroscopic‐QED representation of field correlations in a physically realized, passive, dispersive, and locally equilibrated transformation medium. It underpins the transformation rules in Section 3 and the reduced Casimir‐pressure construction in Section 4.

### 2.9 Local Heat and Entropy Production

The local heat‐generation rate per physical volume is

(10) $${\dot{Q}}^{\prime }\left(r,\omega \right)=\omega \;\text{Im}\;\left[\varepsilon^{\prime \;ij}\left(r,\omega \right)\right]\;{\langle E_{i}^{\prime }E_{j}^{\prime }\rangle }_{T},$$

Moreover, the entropy‐production rate is

(11) $${\dot{S}}^{\prime }\left(r\right)=\int_{0}^{\infty }d\omega \frac{{\dot{Q}}^{\prime }\left(r,\omega \right)}{T_{\text{eff}}\left(r\right)}$$

Using the transformation rules for $\varepsilon^{\prime },\mu^{\prime }$ and $G^{\prime }$ and the metric weight $\sqrt{|g|}$, these reproduce the metric scaling laws in Sec. 3 (local amplification under compression, dilution under expansion, with conserved total integrals).

### 2.10 Gauge Choice and Boundary Terms

All results above are gauge‐independent; the Lorenz gauge is used for definiteness. In planar Casimir geometries, surface terms generated by partial integrations cancel upon imposing standard continuity conditions on tangential fields. The remaining Hamiltonian density is bulk‐covariant as in Equation (4).

### 2.11 Summary and Outlook

This section establishes thermofield quantization in a curved optical metric as the theoretical backbone of QTTO. TFD provides the quantum–statistical basis, whereas metric embedding promotes it to a covariant field theory where geometry, loss, and temperature are inseparably coupled. The metric‐dependent Hamiltonian density [Equation (4)] and the metric‐FDT [Equation (9)] are the two pillars linking microscopic fluctuations to macroscopic thermodynamics. In this sense, QTTO is not a phenomenological extension of TFD but a unified formalism that generalizes quantum electrodynamics in media and transformation optics to include thermodynamic curvature. Section 3 uses these operator‐level results to derive explicit transformation laws for energy, dissipation, and entropy, laying the groundwork for the Casimir pressure law in Section 4.

Dissipation in QTTO is not an external perturbation to a lossless transformation‐optics design; it is incorporated at the level of the metric‐weighted thermofield Hamiltonian, where absorption, noise, heat generation, and entropy production are linked by the transformed fluctuation‐dissipation relation. This construction reduces to standard macroscopic QED in the flat‐metric limit (|detΛ|=1) and provides a covariant generalization for curved optical spaces.

## 3 Transformation of Fluctuation‐Related Energy and Entropy Measures

### 3.1 Metric‐Dependent Fluctuation Energy Density

Quantities in the implemented dissipative transformation medium. These quantities should not be interpreted as thermodynamic resources generated by a coordinate transformation alone. They are local macroscopic‐QED observables obtained from the transformed field correlations, material response, and metric‐weighted Green tensor under the assumptions stated in Section 2.3. The physical bath temperature remains T, whereas $T_{\text{eff}}$ is used only as the effective statistical parameter appearing in the local representation of the transformed fluctuation spectrum.

Starting from the Hamiltonian density in the curved optical metric, Equation (4),

$$H_{T}^{\prime }=\frac{\left|g\right|}{2}\left(E_{i}^{\prime }\;\varepsilon^{\prime ij}E_{j}^{\prime }+B_{i}^{\prime }\;\mu^{\prime ij}B_{j}^{\prime }\right)$$

The mean energy density at finite temperature follows from the thermofield expectation value (cf. Equation (5)):

$$U^{\prime }\left(r,T\right)=\frac{\hbar }{\pi }\;\int_{0}^{\infty }\omega \;\cot\;h\left(\frac{\hbar \omega }{2k_{B}T_{\text{eff}}\left(r\right)}\right)\text{Im}\left[\text{Tr}\varepsilon^{\prime }\left(r,\omega \right)G^{\prime }\left(r,r;\omega \right)\right]d\omega$$

Applying the transformation rules $\varepsilon^{\prime ij}={\left|\det\;\Lambda \right|}^{-1}\Lambda_{m}^{i}\Lambda_{n}^{j}\varepsilon^{mn}$ and $|g|={\left|\det\;\Lambda \right|}^{-1}$, and assuming local isotropy $\varepsilon_{mn}=\varepsilon \delta_{mn}$, yields the geometric scaling law

$$U^{\prime }=U_{0}\;{\left|\det\;\Lambda \right|}^{-1}F_{E}\left(\Lambda \right),F_{E}\left(\Lambda \right)=\frac{\left(\Lambda E\right)\;\cdot \;\left(\Lambda E\right)}{E\;\cdot \;E}$$

Thus, in a physically implemented transformation medium, geometric compression (|detΛ|<1) increases the local weighting of fluctuation energy density, whereas expansion (|detΛ|>1) decreases it. This should be understood as a redistribution of the local field‐correlation measure induced by the implemented optical metric, not as the creation or destruction of electromagnetic energy by a coordinate transformation. Because the metric‐weighted volume element transforms consistently with the local density, the corresponding integrated fluctuation energy remains invariant within the stated local‐equilibrium approximation. Note that $d^{3}x^{\prime }=\left|\det\;\Lambda \right|\;d^{3}x$, the total energy remains invariant:

$$\int U^{\prime }d^{3}x^{\prime }=\int U_{0}d^{3}x=\text{const}.$$

### 3.2 Metric‐Dependent Heat‐Generation Density

Within macroscopic quantum electrodynamics, the local rate of heat generation per unit volume is

$$\dot{Q}=\omega \;\text{Im}\left[\varepsilon_{ij}\left(\omega \right)\right]\;{\langle E_{i}E_{j}\rangle }_{T}.$$

Under the coordinate transformation, and using the same metric scaling of fields and tensors as above, one obtains

$${\dot{Q}}^{\prime }={\dot{Q}}_{0}\;{\left|\det\;\Lambda \right|}^{-1}\;F_{E}\left(\Lambda \right).$$

Hence, within the local macroscopic‐QED description, the heat‐generation density and the fluctuation energy density acquire analogous Jacobian‐dependent weights. Compression of the implemented optical metric increases the local dissipative weighting, whereas expansion decreases it. This statement refers to the spatial organization of absorption‐related fluctuation observables in the realized medium; it does not imply that a passive coordinate transformation by itself generates heat.

### 3.3 Entropy‐Related Response and Global Balance

Using the local relation between absorption‐related heat generation and the effective statistical weighting parameter introduced in Equation (6), we define a local entropy‐related response measure as

$${\dot{S}}^{\prime }=\frac{\omega }{T_{\text{eff}}}\;\text{Im}\left[\varepsilon_{ij}^{\prime }\left(\omega \right)\right]{\langle E_{i}^{\prime }E_{j}^{\prime }\rangle }_{T}\;|g|={\dot{S}}_{0}F_{E}\left(\Lambda \right)$$

Here, ${\dot{S}}^{\prime }$ should be understood as an entropy‐related response associated with dissipative fluctuation correlations in the implemented medium. It is not entropy production generated by the coordinate mapping itself. Real entropy production is tied to absorption, reservoir dynamics, and nonequilibrium processes in the physical material. The role of the optical metric is to redistribute the local statistical weighting of these dissipative fluctuation contributions through the transformed material response, mode normalization, and Green‐tensor correlations.

Although this local entropy‐related measure varies with the mapping, its metric‐weighted integral over the physical space remains invariant within the local‐equilibrium approximation,

$$S_{\text{tot}}^{\prime }=\int {\dot{S}}^{\prime }d^{3}x^{\prime }=\int {\dot{S}}_{0}d^{3}x.$$

Therefore, QTTO preserves the global balance of the fluctuation‐related entropy measure. The implemented optical geometry redistributes the local weighting of entropy‐related correlations, but it does not create entropy from geometry alone and does not modify the second law of thermodynamics.

### 3.4 Analytical Example: Radial Mapping $r^{\prime }=r^{\alpha }$

To illustrate the preceding scaling rules, we consider a simple isotropic radial mapping of the form.

$r^{\prime }=r^{\alpha }$ with, $\left|\det\;\Lambda \right|=\alpha \;r^{2\left(\alpha -1\right)}$ where α controls the degree of radial compression or expansion. For α<1, the mapping locally compresses the implemented optical geometry, whereas for α>1, it produces an effective expansion. The identity transformation is recovered for α=1.

For this mapping, the local Jacobian factor modifies the fluctuation‐related energy density and the entropy‐related response measure through the same effective geometric weighting. In normalized form, we may write

$$\frac{u^{\prime }\left(r\right)}{u_{0}\left(r\right)}=F_{E}\left(\alpha ,r\right),\frac{{\dot{S}}^{\prime }\left(r\right)}{{\dot{S}}_{0}\left(r\right)}=F_{E}\left(\alpha ,r\right)$$

where $u^{\prime }\left(r\right)$ denotes the transformed fluctuation energy density, ${\dot{S}}^{\prime }\left(r\right)$ denotes the entropy‐related response measure defined in Section 3.3, and $F_{E}\left(\alpha ,r\right)$ is the Jacobian‐dependent scaling factor associated with the implemented optical metric. For the radial mapping considered here, this factor may be expressed as

$$F_{E}\left(\alpha ,r\right)=\frac{1}{\alpha }r^{-2\left(\alpha -1\right)}$$

Thus,

$$\frac{U^{\prime }\left(r\right)}{U_{0}\left(r\right)}=\frac{{\dot{S}}^{\prime }\left(r\right)}{{\dot{S}}_{0}\left(r\right)}=\frac{1}{\alpha }r^{-2\left(\alpha -1\right)}$$

This example shows that compression and expansion do not create thermodynamic resources. Rather, they change the local statistical weighting of fluctuation‐related observables in the physically implemented transformation medium. The corresponding interpretation is summarized in Table 1.

**TABLE 1:** Effect of geometric compression and expansion on local fluctuation‐related energy density and entropy‐related response in an implemented transformation‐optical medium.

Mapping type	α	Energy and entropy density	Physical meaning
Compression	*α* < 1	↑ Enhanced	Enhanced local fluctuation weighting and entropy‐related response
Identity	*α* = 1	Constant	Unchanged local fluctuation weighting
Expansion	*α* > 1	↓ Decreased	Reduced local fluctuation weighting and entropy‐related response

*Note:* The parameter *α* characterizes the radial mapping, with compression (*α* < 1), identity (*α* = 1), and expansion (*α* > 1).

Both $U^{\prime }\left(r\right)$ and ${\dot{S}}^{\prime }\left(r\right)$ follow the same normalized scaling in this illustrative isotropic mapping. This common behavior reflects the fact that both quantities are constructed from the same transformed field correlations and material response. It should not be interpreted as physical heating, cooling, or entropy generation by the coordinate mapping itself. Any real heat generation or entropy production remains associated with absorption and reservoir dynamics in the dissipative material implementation.

### 3.5 Physical Interpretation

The relations presented in Sections 3.1, 3.2, 3.3, 3.4 should be interpreted as transformation rules for fluctuation‐related observables in a physically implemented dissipative transformation medium.

$$|\det\;\Lambda {|}^{-1}\;↔\;\text{local}\;\text{amplification}\;\text{of}\;\left(U,\dot{Q},\dot{S}\right),$$

$$T_{\text{eff}}=T/\left|\det\;\Lambda \right|\;↔\;\text{metric}‐\text{induced}\;\text{temperature}\;\text{shift}.$$

They do not imply that a coordinate transformation alone produces local heating, cooling, or entropy generation. Rather, the implemented optical metric changes the local normalization of modes, the density of electromagnetic states, and the Green‐tensor correlations that enter the macroscopic‐QED fluctuation spectrum.

Within this interpretation, geometric compression may be described as increasing the local statistical weight of quantum and thermal fluctuations, whereas expansion decreases that weight. This language is analogous to a redshift or blueshift of modal occupation, but it remains an effective description of field correlations in the realized medium. The actual thermodynamic bath temperature remains T, and any real dissipation or entropy production arises from the lossy material response and its reservoir degrees of freedom, not from the coordinate mapping itself.

Thus, the main result of this section is not that geometry creates thermodynamic resources, but that a transformation‐optical implementation provides a controlled way to reorganize fluctuation‐related energy, heat‐generation, and entropy‐response measures while preserving global balance laws.

### 3.6 Transition to the Casimir Application

The results of Section 3 provide the local fluctuation‐scaling structure used in the Casimir application below. They show how, under the stated assumptions, an implemented optical metric reorganizes the local statistical weights entering macroscopic‐QED correlation functions. The Casimir pressure provides a useful test case because it depends directly on the same fluctuation spectrum, material response, and boundary conditions. Therefore, Section 4 applies the QTTO construction to a planar dissipative cavity as a controlled example. The resulting pressure law should be understood as a reduced asymptotic description of the quantum‐to‐classical crossover, not as a replacement for the full Lifshitz–Matsubara calculation.

## 4 Casimir Pressure Under Quantum Thermodynamic Transformation Optics (QTTO)

The Casimir effect is the mechanical manifestation of electromagnetic field fluctuations constrained by material response, geometry, temperature, and boundary conditions. In dissipative metamaterials, these fluctuations are described most generally by macroscopic quantum electrodynamics and, for planar equilibrium configurations, by the finite‐temperature Lifshitz–Matsubara formulation. The purpose of the present section is not to replace that full spectral theory. Instead, we use the QTTO construction to obtain a reduced finite‐temperature pressure law that organizes the Casimir response of an implemented transformation medium into two physically identifiable asymptotic branches: a vacuum‐dominated branch and a thermally dominated branch.

This distinction is important. The pressure expression derived below should be understood as an effective asymptotic interpolation that preserves the correct limiting structure and provides a compact representation of the quantum‐to‐classical crossover. It is not an exact point‐by‐point substitute for the full Lifshitz–Matsubara summation, which retains the complete frequency‐dependent spectral information of the dispersive cavity. Therefore, the role of the QTTO expression is to isolate how the implemented optical metric modifies the reduced thermodynamic organization of the pressure, whereas the full Lifshitz calculation remains the reference benchmark for quantitative validation.

Within this interpretation, the pressure is decomposed into a zero‐temperature vacuum branch and a high‐temperature thermal branch. These branches are then connected by a smooth crossover weighting function. The detailed branch construction is given in Supporting Information S1: Appendix A, the statistical interpretation is discussed in Supporting Information S1: Appendix C, and the reproducible comparison with the finite‐temperature Lifshitz benchmark is provided in Supporting Information S1: Appendix D. Additional parameter‐space illustrations of the reduced QTTO law are collected in Supporting Information S1: Appendix B.

### 4.1 Quantum Limit: Metric‐Dependent Zero‐Point Pressure

We begin with the zero‐temperature contribution. In the limit T→0, the thermofield occupation vanishes and only zero‐point modes remain. Starting from the metric‐weighted Hamiltonian density derived in Section 2, the Casimir pressure is obtained from the vacuum expectation value of the stress tensor and may be referenced to the standard parallel‐plate vacuum pressure

$$P_{0}=-\frac{\pi^{2}\hbar c}{240\;d^{4}}$$

where d is the plate separation. Under a general coordinate transformation with Jacobian Λ, the transformed optical metric modifies the vacuum modal spectrum. For an anisotropic deformation characterized by longitudinal and transverse scale factors $s_{z}$ and $s_{⊥}$, we define the dimensionless compression ratio $\rho =\frac{s_{z}}{s_{⊥}}.$ The transformed zero‐point branch then scales as

(12) $$\frac{P_{q}^{\prime }}{P_{0}}=\rho^{2}.$$

Thus, within the reduced branch description, longitudinal compression increases the normalized vacuum contribution, whereas longitudinal expansion decreases it. This result should be interpreted as the zero‐temperature asymptotic scaling of the implemented optical metric, after the transformed material response and boundary conditions have been specified. It is not a statement that a coordinate transformation alone changes the physical vacuum pressure. Equation (12) provides the vacuum‐branch input to the reduced QTTO interpolation; the corresponding operator‐level motivation is summarized in Supporting Information S1: Appendix A.

### 4.2 Classical (High‐Temperature) Limit

In the opposite regime, the field correlations are dominated by thermally populated modes. In the local effective representation introduced in Section 2, the transformed Bose weight is expressed in terms of the operational statistical parameter $T_{\text{eff}}$. The physical reservoir remains at the bath temperature T, whereas $T_{\text{eff}}$ summarizes how the implemented optical metric modifies the local fluctuation spectrum through the transformed mode normalization, density of states, and Green tensor.

In the high‐temperature limit, $k_{B}T_{\text{eff}}\gg \hbar \omega^{\prime }$, the Bose–Einstein occupation factor reduces to the Rayleigh–Jeans form,

$$n_{B}\left(\omega^{\prime },T_{\text{eff}}\right)\approx \frac{k_{B}T_{\text{eff}}}{\hbar \omega^{\prime }}$$

Therefore, the thermally dominated branch must retain an explicit linear dependence on $T_{\text{eff}}$. This requirement is imposed in the reduced QTTO pressure law so that the classical branch remains consistent with the high‐temperature Lifshitz–Matsubara asymptote. For the anisotropic scaling considered here, the normalized thermal branch is written as

(13) $$\frac{P_{\text{cl}}^{\prime }\left(T\right)}{P_{0}}=\left(\frac{T_{\text{eff}}}{T_{⋆}}\right)s_{⊥}^{-4}$$

Here, $s_{⊥}$ denotes the transverse scale factor and $T_{⋆}$ is a cavity reference crossover scale. The quantity $T_{⋆}$ is not a second effective temperature. It is introduced only because the finite‐temperature branch is normalized by the zero‐temperature reference pressure $P_{0}$. Physically, $T_{⋆}$ characterizes the temperature scale at which the thermal contribution becomes comparable to the vacuum branch for the specific dispersive cavity under consideration.

Therefore, this classical branch should be interpreted as an asymptotic component of the reduced pressure description, not as an independent first‐principles expression replacing the full Matsubara summation. The derivation of this branch from the Rayleigh–Jeans limit is given explicitly in Supporting Information S1: Appendix A, whereas its ensemble‐level interpretation is discussed in Supporting Information S1: Appendix C.

### 4.3 Unified Reduced QTTO Pressure Law

The finite‐temperature expression is constructed by combining the zero‐temperature vacuum branch and the high‐temperature thermal branch through a smooth crossover function. This construction is deliberately reduced: it does not evaluate the full Matsubara spectrum at each temperature, but instead represents the gradual transfer of pressure weight from the vacuum‐dominated sector to the thermally dominated sector. The resulting expression is therefore controlled by its two asymptotic limits and by crossover parameters that encode the dominant spectral scale of the cavity. The detailed branch construction and operator‐level motivation are given in Supporting Information S1: Appendix A, whereas Supporting Information S1: Appendix C provides the corresponding statistical‐field interpretation of the weighting function. The numerical benchmark against the finite‐temperature Lifshitz–Matsubara calculation, including the normalization procedure and parameter choices, is presented in Supporting Information S1: Appendix D.

We introduce the dimensionless crossover variable, $\tau =\frac{T_{\text{eff}}}{T_{⋆}}$, and the smooth weighting function, $W\left(\tau \right)=1-\exp\left(-\tau^{\nu }\right)$, where ν controls the sharpness of the quantum‐to‐classical crossover. The reduced QTTO pressure law is then written as

(14) $$\frac{P^{\prime }\left(T\right)}{P_{0}}=\left[1-W\left(\tau \right)\right]\rho^{2}+W\left(\tau \right)\left(\frac{T_{\text{eff}}}{T_{⋆}}\right)s_{⊥}^{-4}$$

Here, $P_{0}$ is the zero‐temperature reference pressure of the corresponding physical cavity, $\rho =s_{z}/s_{⊥}$ is the longitudinal‐to‐transverse compression ratio, $s_{⊥}$ is the transverse scale factor, and $T_{⋆}$ is a cavity reference crossover temperature. The scale $T_{⋆}$ is not a second effective temperature and should not be confused with $T_{\text{eff}}$. Rather, $T_{⋆}$ characterizes the spectral onset of the thermal contribution after the pressure has been normalized by $P_{0}$. The two limiting regimes follow directly from the behavior of the weighting function,

$$\tau \ll 1\;\Rightarrow \;W\to 0\;\Rightarrow \;\frac{P^{\prime }\left(T\right)}{P_{0}}\to \rho^{2},$$

$$\tau \gg 1\;\Rightarrow \;W\to 1\;\Rightarrow \;\frac{P^{\prime }\left(T\right)}{P_{0}}\to \left(\frac{T_{\text{eff}}}{T_{⋆}}\right)s_{⊥}^{-4}$$

These limits show explicitly that the reduced pressure law preserves the zero‐temperature vacuum branch and the Rayleigh–Jeans thermal branch. The interpolation between them is controlled by W(τ), rather than by a full evaluation of the Matsubara spectrum at each temperature.

In the identity‐mapping limit, ∣detΛ∣=1, ρ=1, and $s_{⊥}=1$. In this case $T_{\text{eff}}=T$, and Equation (14) reduces to

(15) $$\frac{P^{\prime }\left(T\right)}{P_{0}}=\left[1-W\left(T/T_{⋆}\right)\right]+W\left(T/T_{⋆}\right)\frac{T}{T_{⋆}}$$

This identity‐mapping limit now exhibits the proper finite‐temperature variation instead of collapsing to a constant. It resolves the internal inconsistency of the earlier compact form and makes the pressure law compatible with the thermofield occupation structure used in QTTO.

The principal predictive content of QTTO is that the finite‐temperature Casimir pressure in a transformed dispersive cavity separates into two asymptotic sectors with distinct geometric control: a vacuum branch governed by $\rho^{2}$ and a thermally dominated branch governed by $\left(T_{\text{eff}}/T_{⋆}\right)s_{⊥}^{-4}$, connected by a smooth crossover law. In this sense, the implemented optical metric controls the relative weighting of the vacuum and thermal sectors and can shift the apparent quantum‐to‐classical crossover of the reduced pressure response. This prediction should be understood within the reduced asymptotic framework of Equation (14), not as a point‐by‐point replacement for the full Lifshitz–Matsubara theory.

Therefore, Equation (14) is not universal in the same sense as the Lifshitz–Matsubara formula. Its validity is restricted to passive, locally equilibrated, and dispersive transformation media for which the optical metric varies slowly enough that a local macroscopic‐QED description is meaningful. Intermediate‐temperature deviations from the full Lifshitz result are expected whenever detailed spectral features of the material response cannot be represented by the two‐branch reduction. The determination of $T_{⋆}$, the fitting of ν, and the range of validity of this reduced description are specified in the following subsection.

### 4.4 Physical Interpretation and Range of Validity

Equation (14) shows that the Casimir response in QTTO is governed by the competition between two asymptotic fluctuation sectors: a vacuum‐dominated sector controlled by $\rho^{2}$, and a thermally dominated sector controlled by $\left(T_{\text{eff}}/T_{⋆}\right)s_{⊥}^{-4}$. This is the principal physical content of the reduced pressure law: the implemented optical metric changes the relative weight of the vacuum and thermal sectors and thereby shifts the quantum‐to‐classical crossover of the Casimir response. This interpretation should be understood within the reduced asymptotic framework of Equation (14), not as a point‐by‐point replacement for the full Lifshitz–Matsubara theory.

In the low‐temperature regime, the pressure is dominated by the zero‐point branch. Compression of the implemented optical metric increases the local modal weighting and enhances the normalized vacuum contribution, so the reduced Casimir response reflects geometric confinement of the quantum fluctuation spectrum. In the high‐temperature regime, thermally populated modes dominate; $T_{\text{eff}}$ parametrizes their effective statistical weight, whereas $s_{⊥}^{-4}$ gives the geometric modulation of their stress contribution. This should not be interpreted as physical heating or cooling by a coordinate transformation. The physical reservoir remains at the bath temperature T; the transformation medium reorganizes the fluctuation spectrum through mode normalization, density of states, and Green‐tensor correlations. The crossover parameters specify the reduced character of Equation (14). The scale $T_{⋆}$ is not a second effective temperature, but a cavity‐dependent reference scale for the onset of the thermal contribution in the normalized pressure. The exponent ν controls the smoothness of the transition between the vacuum and thermal branches. The statistical‐field interpretation of this reduced two‐sector structure is discussed in Supporting Information S1: Appendix C, whereas the numerical calibration of $T_{⋆}$ and ν against the corresponding finite‐temperature Lifshitz–Matsubara benchmark is described in Supporting Information S1: Appendix D.

Therefore, Equation (14) should be read as a controlled reduced representation of the finite‐temperature Casimir crossover, not as an exact point‐by‐point substitute for the full Lifshitz–Matsubara summation. Its validity is restricted to passive, locally equilibrated, and dispersive transformation media for which the optical metric varies slowly enough that a local macroscopic‐QED description remains meaningful. When sharp resonances, strong nonlocality, nonequilibrium temperature gradients, or detailed geometry‐specific scattering dominate the spectrum, the full Lifshitz–Matsubara or Green‐tensor calculation remains necessary.

### 4.5 Numerical Validation

To validate the revised QTTO pressure law, we compare Equation (14) with the finite‐temperature Lifshitz–Matsubara pressure for a dispersive Drude–Lorentz magnetodielectric cavity. The comparison is performed using the same material response in both calculations, so that any difference between the two curves reflects the reduced nature of the QTTO interpolation rather than a change of physical model. The numerical protocol, material parameters, Matsubara summation, and normalization procedure are given in Supporting Information S1: Appendix D. All material parameters, the plate separation, the normalization prescription, and the crossover parameters used in the comparison are stated explicitly in Supporting Information S1: Appendix D to make the benchmark reproducible.

A crucial point in this comparison is the normalization by the zero‐temperature reference pressure $P_{0}$. In the revised calculation, $P_{0}\equiv P\left(d,0\right)$ is obtained from the corrected zero‐temperature Lifshitz integral for the same dispersive cavity. The finite‐temperature Lifshitz pressure $P_{\text{Lif}}\left(T\right)$ is then evaluated from the corresponding Matsubara summation and normalized by this same $P_{0}$. This ensures that the normalized Lifshitz result satisfies the correct low‐temperature limit, $\frac{P_{\text{Lif}}\left(T\right)}{P_{0}}\to 1\text{as}T\to 0.$ This corrected normalization is essential because the reduced QTTO law is also written in terms of the same reference pressure $P_{0}$.

For the benchmark calculation, we consider the identity‐mapping limit,

$$\rho =1,s_{⊥}=1,|\det\;\Lambda |=1$$

So that $T_{\text{eff}}=T.$ This choice isolates the temperature‐driven crossover without adding geometric anisotropy. The revised QTTO prediction is then evaluated using

$$\nu =2.5,T_{⋆}=4.2\times 10^{3}\;K$$

Here, $T_{⋆}$ is interpreted as the cavity reference crossover scale governing the onset of thermal dominance in the normalized pressure law, whereas ν controls the smoothness of the crossover between the vacuum and thermal branches.

The comparison shows that the revised QTTO law reproduces the correct normalized quantum limit at low temperature and captures the expected thermal growth at high temperature. In the intermediate crossover region, moderate deviations remain because the full Lifshitz–Matsubara calculation retains detailed spectral information from all Matsubara frequencies, whereas Equation (14) represents this structure through a two‐branch reduced crossover function. These deviations are therefore expected and are consistent with the interpretation of Equation (14) as a reduced asymptotic pressure law rather than an exact point‐by‐point substitute for Lifshitz theory.

Therefore, the numerical validation supports the revised QTTO formulation in the intended sense. It confirms that the pressure law is anchored to the corrected zero‐temperature Lifshitz normalization, preserves the proper vacuum branch, restores the explicit high‐temperature thermal dependence, and captures the overall quantum‐to‐classical crossover with a compact analytical expression. The full Lifshitz–Matsubara formulation remains the quantitative benchmark, whereas the QTTO law provides a reduced thermodynamic representation of the same crossover structure.

### 4.6 Implications for Planar and Structured Casimir Geometries

The Casimir effect, often viewed as a purely vacuum‐fluctuation phenomenon, acquires a broader interpretation within QTTO as the observable stress response of a quantum and thermal electromagnetic fluctuations in an implemented optical metric. In the reduced formulation developed above, the vacuum branch reflects the geometric scaling of zero‐point fluctuations, whereas the thermal branch reflects thermally populated modes weighted by $T_{\text{eff}}$ and modulated by transverse metric scaling.

The revised pressure law makes this structure explicit. QTTO does not replace the full Lifshitz–Matsubara theory in every spectral detail, but provides a compact reduced representation of the transition between vacuum‐dominated and thermally dominated Casimir regimes. Its value lies in exposing how geometry, dissipation, and statistical field occupation jointly organize the finite‐temperature pressure response in a transformed dispersive cavity.

In this sense, the Casimir force is described as the outcome of a covariant redistribution of fluctuation‐related energy measures and thermally weighted modal occupation in the implemented optical metric. This interpretation remains consistent with the standard Lifshitz–Matsubara benchmark, while highlighting the reduced thermodynamic structure underlying the crossover between the quantum and classical regimes.

A natural extension of the present framework is to laterally structured geometries, such as grating Casimir configurations, where transformation‐induced optical metrics, dissipation, and thermal modal structure may interact in a more complex way. Such systems lie beyond the scope of the present work, whose purpose is to establish the QTTO formalism in the analytically controlled planar cavity geometry.

## 5 Conclusions

In conclusion, this work develops quantum thermodynamic transformation optics (QTTO) as a metric‐based framework for describing quantum and thermal electromagnetic fluctuations in physically implemented dissipative transformation media. The central idea is that a transformation‐optical mapping, when realized through dispersive and lossy material tensors together with the required reservoir degrees of freedom and boundary conditions, reorganizes the statistical structure of field correlations. Thus, QTTO should not be interpreted as assigning thermodynamic effects to a passive coordinate transformation alone, but as an effective macroscopic‐QED description of fluctuation observables in implemented optical metrics.

Within this framework, we derived transformation rules for fluctuation‐related energy density, heat‐generation density, and entropy‐related response. These quantities acquire Jacobian‐dependent local weights under the implemented optical metric, so that compression enhances the local statistical weighting of the fluctuation spectrum whereas expansion reduces it. The corresponding global balance laws remain preserved within the local‐equilibrium approximation. In this sense, QTTO does not introduce artificial sources of energy, heat, or entropy; rather, it provides a covariant description of how existing quantum and thermal fluctuations are redistributed by the physical transformation medium.

A key element of the formulation is the metric‐dependent effective statistical temperature $T_{\text{eff}}$. This quantity is not a physical bath temperature and does not imply local heating or cooling of the material. Instead, it is an operational parameter that appears when the transformed fluctuation spectrum is written in the standard thermofield/FDT form. Its role is to parametrize the Jacobian‐dependent statistical weighting of quantum and thermal field correlations in a passive, locally equilibrated, and dispersive medium.

As a concrete application, we reformulated the finite‐temperature Casimir pressure in a transformed dissipative cavity. The resulting QTTO pressure law separates the response into a vacuum‐dominated branch governed by $\rho^{2}$ and a thermally dominated branch governed by $\left(T_{\text{eff}}/T_{⋆}\right)s_{⊥}^{-4}$, connected by a smooth crossover function. This expression should be understood as a reduced asymptotic representation of the quantum‐to‐classical Casimir crossover, not as an exact point‐by‐point replacement for the full Lifshitz–Matsubara theory. The crossover scale $T_{⋆}$ and exponent ν encode the cavity‐dependent onset and smoothness of the thermal branch and are calibrated against the corresponding Lifshitz benchmark.

The numerical comparison with the finite‐temperature Lifshitz–Matsubara formulation confirms the intended validity of the reduced QTTO law. Using the corrected zero‐temperature Lifshitz normalization, the revised pressure expression reproduces the low‐temperature vacuum limit, captures the high‐temperature thermal trend, and describes the overall crossover structure in compact analytical form. Deviations in the intermediate region are expected because the full Matsubara summation retains detailed spectral information that is compressed in the reduced two‐branch QTTO representation.

To the best of our knowledge, QTTO provides one of the first analytical routes toward a metric‐based thermodynamic framework of quantum fields in dissipative and curved optical media. This opens a path for studying geometry‐controlled fluctuation forces, thermal field correlations, and quantum–thermal response in structured optical environments. Future work may extend the present formulation to nonequilibrium reservoirs, laterally structured Casimir geometries, such as gratings, and experimentally realizable quantum‐thermal photonic and metamaterial systems.

## Author Contributions

**Mohammad Mehdi Sadeghi:** investigation, funding acquisition, writing – original draft, methodology, validation, visualization, writing – review and editing, software, formal analysis, conceptualization, project administration, supervision. **Mustafa Sarisaman:** writing – original draft, writing – review and editing, formal analysis.

## Funding

The authors have nothing to report.

## Supporting information


Supporting Information S1

## Data Availability

The data that support the findings of this study are available from the corresponding author upon reasonable request.

## References

[1] J. B. Pendry , D. Schurig , and D. R. Smith , “Controlling Electromagnetic Fields,” Science 312, no. 5781 (2006): 1780–1782, 10.1126/science.1125907. 16728597

[2] U. Leonhardt , “Optical Conformal Mapping,” Science 312, no. 5781 (2006): 1777–1780, 10.1126/science.1126493. 16728596

[3] H. Chen , C. T. Chan , and P. Sheng , “Transformation Optics and Metamaterials,” Nature Materials 9, no. 5 (2010): 387–396, 10.1038/nmat2743. 20414221

[4] M. M. Sadeghi , S. Li , L. Xu , Bo Hou , and H. Chen , “Transformation Optics With Fabry–Pérot Resonances,” Scientific Reports 5, no. 1 (2015): 8680, 10.1038/srep08680. PMC4345524 25726924

[5] D. Schurig , J. J. Mock , B. J. Justice , et al., “Metamaterial Electromagnetic Cloak at Microwave Frequencies,” Science 314, no. 5801 (2006): 977–980, 10.1126/science.1133628. 17053110

[6] M. Rahm , D. Schurig , D. A. Roberts , S. A. Cummer , D. R. Smith , and J. B. Pendry , “Design of Electromagnetic Cloaks and Concentrators Using Form‐Invariant Coordinate Transformations of Maxwell’s Equations,” Photonics and Nanostructures 6, no. 1 (2008): 87–95, 10.1016/j.photonics.2007.07.013.

[7] M. M. Sadeghi , H. Nadgaran , and H. Chen , “Perfect Field Concentrator Using Zero‐Index Metamaterials and Perfect Electric Conductors,” Frontiers of Physics 9, no. 1 (2014): 90–93, 10.1007/s11467-013-0374-0.

[8] C. Qian , I. Kaminer , and H. Chen , “A Guidance to Intelligent Metamaterials and Metamaterials Intelligence,” Nature Communications 16, no. 1 (2025): 1154, 10.1038/s41467-025-56122-3. PMC11779837 39880838

[9] S. Franke , J. Ren , S. Hughes , and M. Richter , “Fluctuation‐Dissipation Theorem and Fundamental Photon Commutation Relations in Lossy Nanostructures Using Quasinormal Modes,” Physical Review Research 2, no. 3 (2020): 033332, 10.1103/physrevresearch.2.033332.

[10] J. Feist , A. I. Fernández‐Domínguez , and F. J. García‐Vidal , “Macroscopic QED for Quantum Nanophotonics: Emitter‐Centered Modes as a Minimal Basis for Multiemitter Problems,” Nanophotonics 10 (2021): 477, 10.1515/nanoph-2020-0451.

[11] E. Amooghorban , M. Wubs , N. A. Mortensen , and F. Kheirandish , “Electromagnetic Field Quantization and Spontaneous Decay in Arbitrary Inhomogeneous Lossy Media,” Physical Review A 84 (2011): 013806, 10.1103/PhysRevA.84.013806.

[12] F. Kheirandish , E. Amooghorban , and M. Soltani , “Finite‐Temperature Quantization of Electromagnetic Field in Absorbing Media,” Physical Review A 83 (2011): 032507, 10.1103/PhysRevA.83.032507.

[13] E. Amooghorban and M. S. Kheirandish , “Quantum Electrodynamics of Dissipative and Dispersive Media: A Thermofield Approach,” Physical Review A 97 (2018): 043803, 10.1103/PhysRevA.97.043803.

[14] H. Umezawa , H. Matsumoto , and M. Tachiki , Thermo Field Dynamics and Condensed States (North‐Holland, 1982).

[15] Y. Takahashi and H. Umezawa , “Thermo Field Dynamics,” Collective Phenomena 2 (1975): 55.

[16] A. Mann , M. Revzen , H. Umezawa , and Y. Yamanaka , “Relation Between Quantum and Thermal Fluctuations,” Physics Letters A 140 (1989): 475–478, 10.1016/0375-9601(89)90125-4.

[17] P. D. Nation , J. R. Johansson , M. P. Blencowe , and F. Nori , “Colloquium: Stimulating Uncertainty—Amplifying the Quantum Vacuum With Superconducting Circuits,” Reviews of Modern Physics 84 (2012): 1–24, 10.1103/revmodphys.84.1.

[18] E. M. Lifshitz , “The Theory of Molecular Attractive Forces Between Solids,” Soviet Physics Journal of Experimental and Theoretical Physics 2 (1956): 73, https://jetp.ras.ru/cgi‐bin/dn/e_002_01_0073.pdf.

[19] I. E. Dzyaloshinskii , E. M. Lifshitz , and L. P. Pitaevskii , “The General Theory of van der Waals Forces,” Advances in Physics 10 (1961): 165–209, 10.1080/00018736100101281.

[20] G. L. Klimchitskaya , U. Mohideen , and V. M. Mostepanenko , “The Casimir Force Between Real Materials: Experiment and Theory,” Reviews of Modern Physics 81, no. 4 (2009): 1827–1885, 10.1103/revmodphys.81.1827.

[21] K. A. Milton , The Casimir Effect: Physical Manifestations of Zero‐point Energy (World Scientific, 2001).

[22] T. Gong , M. R. Corrado , A. R. Mahbub , C. Shelden , and J. N. Munday , “Recent Progress in Engineering the Casimir Effect—Applications to Nanophotonics, Nanomechanics, and Chemistry,” Nanophotonics 10 (2021): 523, 10.1515/nanoph-2020-0425.

[23] M. Wang , L. Tang , C. Y. Ng , et al., “Strong Geometry Dependence of the Casimir Force Between Interpenetrated Rectangular Gratings,” Nature Communications 12, no. 1 (2021): 600, 10.1038/s41467-021-20891-4. PMC7838308 33500401

[24] B. Munkhbat , A. Canales , B. Küçüköz , D. G. Baranov , and T. O. Shegai , “Tunable Self‐Assembled Casimir Microcavities and Polaritons,” Nature 597, no. 7875 (2021): 214–219, 10.1038/s41586-021-03826-3. 34497392

[25] Z. Xu , X. Gao , J. Bang , Z. Jacob , and T. Li , “Non‐Reciprocal Energy Transfer Through the Casimir Effect,” Nature Nanotechnology 17, no. 2 (2022): 148–152, 10.1038/s41565-021-01026-8. 34903895

[26] Z. Xu , P. Ju , X. Gao , K. Shen , Z. Jacob , and T. Li , “Observation and Control of Casimir Effects in a Sphere‐Plate‐Sphere System,” Nature Communications 13, no. 1 (2022): 6148, 10.1038/s41467-022-33915-4. PMC9579181 36257958

[27] A. N. Babar , J. Mathew , R. N. Patel , et al., “Self‐Assembled Photonic Cavities With Atomic‐Scale Confinement,” Nature 624, no. 7990 (2023): 57–63, 10.1038/s41586-023-06736-8. PMC10700130 38057568

[28] Y. Hu , X. Wu , H. Liu , and X. Huang , “Casimir Interaction Driven by Hyperbolic Polaritons,” Nanophotonics 13, no. 16 (2024): 2983–2994, 10.1515/nanoph-2024-0065. PMC11501208 39634309

[29] Y. Zhang , H. Zhang , X. Wang , et al., “Magnetic‐Field Tuning of the Casimir Force,” Nature Physics 20, no. 8 (2024): 1282–1287, 10.1038/s41567-024-02521-0.

[30] G. Vagli , T. Tian , F. Naef , et al., “Strong Repulsive Lifshitz–van der Waals Forces on Suspended Graphene,” Nature Communications 16, no. 1 (2025): 7726, 10.1038/s41467-025-63074-1. PMC12365322 40830143

