# Supplementary material for: Quantum Thermodynamic Transformation Optics: A Unified Framework for Energy and Entropy With Application to the Casimir Force in Dissipative Metamaterials
Source: Nanophotonics. 2026 Nov 2;15(21):e70341. doi: 10.1002/nap2.70341 (PMC13726501; doi:10.1002/nap2.70341)
Supplement: Supplementary file 1 [file NAP2-15-e70341-s001.docx]

**Appendix A, B, C and D**

**Appendix A. Derivation of the Revised QTTO Casimir Pressure Law**

The purpose of this appendix is to derive the revised QTTO pressure law used in Sec. 4 from the thermofield Hamiltonian and the modal occupation structure in the transformed optical metric. In the revised formulation, the quantum and classical branches are identified separately and then connected through a smooth thermal weighting function. This construction preserves the geometric zero-temperature scaling, restores the explicit dependence of the classical branch on the effective temperature, and yields a self-consistent reduced thermodynamic interpolation for the Casimir pressure. The revision is necessary because, once the effective temperature enters the thermofield correlators and the fluctuation–dissipation structure, the classical branch must remain explicitly proportional to the thermal occupation factor appearing in the Rayleigh–Jeans limit.

**A.1 Starting Hamiltonian Density**

Within the thermofield formalism, the total Hamiltonian density of the electromagnetic field in a curved optical metric and in the presence of a dispersive–dissipative magnetodielectric medium is written as

$$\begin{matrix} & \mathcal{H}_{\mathrm{TFD}}^{'}=\frac{\sqrt{\mid g\mid}}{2}(\mathbf{E}^{'}\cdot{\bar{\bar{\varepsilon}}}^{'}\cdot\mathbf{E}^{'}+\mathbf{H}^{'}\cdot{\bar{\bar{\mu}}}^{'}\cdot\mathbf{H}^{'})+\mathcal{H}_{\mathrm{res}}^{'}, & & \text{(A1)} \end{matrix}$$

where the primed quantities denote fields in the transformed coordinates, $g$is the effective optical metric induced by the transformation Jacobian $\Lambda$, and $\mathcal{H}_{\mathrm{res}}^{'}$ is the reservoir contribution required for fluctuation–dissipation consistency in the dissipative medium.

The thermofield expectation value of the Hamiltonian is governed by the metric-dependent field correlator, which may be written schematically as

$$\begin{matrix} & \langle\mathcal{H}_{\mathrm{TFD}}^{'}\rangle\propto\int_{0}^{\infty} d\omega\text{ }\hbar\omega\text{ }\coth(\frac{\hbar\omega}{2k_{B}T_{\mathrm{eff}}})\text{ }\mathrm{Im}\text{ }\mathrm{Tr}[{\bar{\bar{\varepsilon}}}^{'}(\omega)\text{ }\mathbf{G}^{'}(\omega)]. & & \text{(A2)} \end{matrix}$$

The relevant temperature entering the transformed thermofield correlator is the effective statistical temperature

$$\begin{matrix} & T_{\mathrm{eff}}(\mathbf{r})=\frac{T}{\left| det\Lambda(\mathbf{r}) \right|} & & \text{(A3)} \end{matrix}$$

This quantity must remain explicit throughout the derivation, because it is the temperature that governs the occupation of transformed modes in QTTO.

**A.2 Pressure from the Thermofield Stress Tensor**

The Casimir pressure is obtained from the normal component of the stress tensor evaluated in the thermofield state,

$$\begin{matrix} & P^{'}(T)=\langle T_{zz}^{'}\rangle. & & \text{(A4)} \end{matrix}$$

At the operator level, this pressure inherits both geometric and thermodynamic dependence. The geometry modifies the transformed modal spectrum and the corresponding mode normalization, while the thermofield state modifies the occupation of those modes. In QTTO these two effects are coupled, but their asymptotic branches can be isolated cleanly and then recombined in reduced analytical form.

**A.3 Quantum Branch: Zero-Point Scaling**

In the limit $T_{\mathrm{eff}}\to0$, the thermal occupation vanishes and only zero-point modes remain. Under an anisotropic transformation characterized by longitudinal and transverse scale factors $s_{z}$ and $s_{\perp}$, we define the dimensionless compression ratio

$$\begin{matrix} & \rho=\frac{s_{z}}{s_{\perp}}. & & \text{(A5)} \end{matrix}$$

The transformed zero-temperature pressure then scales as

$$\begin{matrix} & \frac{P_{q}^{'}}{P_{0}}=\rho^{2}, & & \text{(A6)} \end{matrix}$$

where $P_{0}\equiv P(d,0)$is the zero-temperature Casimir reference pressure of the corresponding physical cavity.

Equation (A6) represents the purely geometric vacuum branch. Longitudinal compression enhances the zero-point stress quadratically, whereas longitudinal expansion reduces it. This branch is the pressure-level analogue of the geometric amplification of local energy density derived in the main text.

**A.4 Classical Branch: Rayleigh–Jeans Limit and Explicit Role of** $\boldsymbol{T}_{\mathbf{eff}}$

At finite temperature, the Bose–Einstein occupation factor of each transformed mode is

$$\begin{matrix} & n_{B}(\omega^{'},T_{\mathrm{eff}})=\frac{1}{\exp(\hbar\omega^{'}/(k_{B}T_{\mathrm{eff}}))-1} & & \text{(A7)} \end{matrix}$$

In the high-temperature regime,

$$k_{B}T_{\mathrm{eff}}\gg\hbar\omega^{'}$$

the occupation reduces to the Rayleigh–Jeans form

$$\begin{matrix} & n_{B}(\omega^{'},T_{\mathrm{eff}})\approx\frac{k_{B}T_{\mathrm{eff}}}{\hbar\omega^{'}}. & & \text{(A8)} \end{matrix}$$

This relation is decisive for the structure of the classical QTTO branch. Since the high-temperature modal occupation is explicitly proportional to $T_{\mathrm{eff}}$, the thermally dominated pressure branch must also retain an explicit linear dependence on $T_{\mathrm{eff}}$. Therefore, the classical pressure cannot be written as a purely geometric quantity. Rather, it must take the general reduced form

$$\begin{matrix} & P_{\mathrm{cl}}^{'}(T)\propto T_{\mathrm{eff}}\times(\text{geometric modulation}). & & \text{(A9)} \end{matrix}$$

Within the present QTTO construction, the geometric modulation of the classical branch is governed by the transverse scale factor $s_{\perp}$, which controls the effective dilution or concentration of thermally populated modes in the transformed optical volume. We therefore write the normalized classical branch as

$$\begin{matrix} & \frac{P_{\mathrm{cl}}^{'}(T)}{P_{0}}=(\frac{T_{\mathrm{eff}}}{T_{\star}})s_{\perp}^{-4}, & & \text{(A10)} \end{matrix}$$

where $T_{\star}$ is a reference crossover scale of the dispersive cavity.

It is important to stress that $T_{\star}$ is not a second effective temperature. The effective statistical temperature of the mapped field remains $T_{\mathrm{eff}}$, as defined in Eq. (A3). The scale $T_{\star}$appears only because the finite-temperature branch is normalized here by the zero-temperature pressure $P_{0}$. Physically, $T_{\star}$ characterizes the spectral onset of the thermal correction in the cavity and is determined by the dominant modal structure of the same dispersive system.

**A.5 Thermal Weighting Function**

To connect the zero-point and classical branches smoothly, we introduce a dimensionless thermal weighting function $W(\tau)$, where

$$\begin{matrix} & \tau=\frac{T_{\mathrm{eff}}}{T_{\star}} & & \text{(A11)} \end{matrix}$$

The role of $W(\tau)$ is to represent the fraction of the total pressure carried by the thermally populated sector of the transformed modal spectrum. A reduced statistical definition may be written as

$$\begin{matrix} & W(\tau)=\frac{\int_{0}^{\infty} d\omega\text{ }n_{B}(\omega,T_{\mathrm{eff}})\text{ }D(\omega)}{\int_{0}^{\infty} d\omega\text{ }[\frac{1}{2}+n_{B}(\omega,T_{\mathrm{eff}})]D(\omega)} & & \text{(A12)} \end{matrix}$$

where $D(\omega)$ denotes the relevant transformed modal density.

In the present QTTO reduction, the detailed spectral integral is represented by the smooth closed form

$$\begin{matrix} & W(\tau)=1-\exp(-\tau^{\nu}), & & \text{(A13)} \end{matrix}$$

where $\nu$controls the sharpness of the quantum-to-classical crossover.

**Figure A1.** Representative thermal weighting function $W(\tau)=1-\exp(-\tau^{\nu})$ used in the revised QTTO pressure law, plotted versus the dimensionless crossover variable $\tau=T_{\mathrm{eff}}/T_{\star}$. The curve smoothly transfers the pressure contribution from the vacuum branch ($W\approx0$) to the thermally dominated branch ($W\approx1$) and provides the reduced crossover structure employed in Eq. (A15).

In the revised interpretation, $W(\tau)$ is not introduced as an exact universal replacement for the full Matsubara sum. Rather, it serves as a reduced thermodynamic crossover function that preserves the correct asymptotic branches and provides a compact analytical representation of the gradual transfer of weight from the zero-point sector to the thermal sector.

**A.6 Final Revised QTTO Pressure Law**

The total QTTO pressure is written as the weighted sum of the quantum and classical branches,

$$\begin{matrix} & P^{'}(T)=[1-W(\tau)]P_{q}^{'}+W(\tau)P_{\mathrm{cl}}^{'}(T). & & \text{(A14)} \end{matrix}$$

Substituting Eqs. (A6), (A10), and (A13), we obtain

$$\begin{matrix} & \frac{P^{'}(T)}{P_{0}}=[1-W(\tau)]\rho^{2}+W(\tau)(\frac{T_{\mathrm{eff}}}{T_{\star}})s_{\perp}^{-4}, \tau=\frac{T_{\mathrm{eff}}}{T_{\star}}, W(\tau)=1-\exp(-\tau^{\nu}). & & \text{(A15)} \end{matrix}$$

This is the revised QTTO pressure law used in Sec. 4 of the main text.

Its two asymptotic limits follow immediately:

$$\begin{matrix} & \tau\ll1\text{ }\Rightarrow\text{ }W\to0\text{ }\Rightarrow\text{ }\frac{P^{'}(T)}{P_{0}}\to\rho^{2}, & & \text{(A16)} \end{matrix}$$

$$\begin{matrix} & \tau\gg1\text{ }\Rightarrow\text{ }W\to1\text{ }\Rightarrow\text{ }\frac{P^{'}(T)}{P_{0}}\to(\frac{T_{\mathrm{eff}}}{T_{\star}})s_{\perp}^{-4}. & & \text{(A17)} \end{matrix}$$

Thus, the revised law preserves the geometric vacuum branch, restores the explicit linear temperature dependence required by the Rayleigh–Jeans regime, and yields a self-consistent thermodynamic interpolation between the two branches.

**A.7 Illustration of the Branch Interpolation**

Figure A2 illustrates the construction of the revised QTTO pressure law in the identity-mapping case $(\rho=1,\text{ }\text{}s_{\perp}=1)$. In this representation, the constant quantum branch, the temperature-dependent classical branch, and the total weighted pressure are shown separately as functions of $\tau=T_{\mathrm{eff}}/T_{\star}$. The figure makes clear how the crossover function $W(\tau)$ gradually transfers the pressure contribution from the vacuum-dominated regime to the thermally dominated regime.

**Figure A2.** Illustration of the revised QTTO pressure interpolation as a function of $\tau=T_{\mathrm{eff}}/T_{\star}$for the identity-mapping case $(\rho=1,\text{ }s_{\perp}=1)$. The constant quantum branch, the temperature-dependent classical branch, and the total weighted pressure are shown separately to visualize how the crossover function $W(\tau)$transfers the pressure contribution from the vacuum-dominated regime to the thermally dominated regime.

This branch-based visualization is useful because it shows explicitly that the revised QTTO law is not a single empirical fit, but the smooth blending of two physically identifiable asymptotic contributions.

**A.8 Physical Meaning of the Revised Crossover Scale**

In the earlier compact formulation, the crossover scale was tied too directly to a bare material frequency scale. The corrected numerical comparison with the finite-temperature Lifshitz theory shows that the thermal crossover of the Casimir pressure in a dispersive cavity is governed not only by the microscopic material resonance, but also by the cavity separation and the dominant spectral content of the modes contributing to the pressure. For this reason, the revised QTTO formulation uses $T_{\star}$ as a cavity reference crossover scale rather than identifying the crossover uniquely with the plasma temperature.

This point is important for physical interpretation. The effective temperature $T_{\mathrm{eff}}$remains the quantity that enters the thermofield correlators and determines thermal population. The scale $T_{\star}$only provides the natural normalization for the onset of thermal dominance in the pressure law. The two quantities therefore have distinct physical roles and should not be conflated.

**Summary of Appendix A**

Starting from the thermofield Hamiltonian in a curved optical metric, we identified two asymptotic pressure branches. The zero-point branch is purely geometric and scales as

$$\frac{P_{q}^{'}}{P_{0}}=\rho^{2}.$$

The high-temperature branch follows from the Rayleigh–Jeans limit of the Bose–Einstein occupation factor and therefore retains an explicit dependence on the metric-induced effective temperature, yielding

$$\frac{P_{\mathrm{cl}}^{'}(T)}{P_{0}}=(\frac{T_{\mathrm{eff}}}{T_{\star}})s_{\perp}^{-4}.$$

By introducing a smooth crossover weighting function $W(\tau)$, with $\tau=T_{\mathrm{eff}}/T_{\star}$, we obtained the revised QTTO pressure law in Eq. (A15), identical to Eq. (4.3) in the main text. The resulting expression should be understood as a self-consistent reduced thermodynamic interpolation that preserves the correct quantum and classical asymptotic structure of the Casimir response in the transformed optical metric.

**Appendix B. Numerical Illustration of the Revised QTTO Pressure Law in a Drude–Lorentz Magnetodielectric Cavity**

**Purpose and Outline**

The purpose of this appendix is to illustrate numerically the behavior of the revised QTTO pressure law for a dispersive and dissipative magnetodielectric cavity described by Drude–Lorentz constitutive functions. In contrast to Appendix D, whose primary role is to benchmark the revised QTTO law directly against the finite-temperature Lifshitz–Matsubara calculation, the present appendix is intended to visualize how the revised QTTO pressure responds to material dispersion, geometric anisotropy, and thermal crossover within the reduced analytical framework itself.

The appendix serves three purposes. First, it presents the material dispersion model used throughout the manuscript on the imaginary-frequency axis. Second, it shows the corrected zero-temperature Lifshitz reference pressure $P_{0}(d)$, which provides the normalization used in the revised QTTO law. Third, it illustrates the behavior of the revised QTTO pressure under variations of temperature and geometric scaling parameters.

All numerical results in this appendix are therefore interpretive rather than exhaustive: they are intended to clarify the structure of the revised QTTO model and its dependence on the parameters entering Eq. (4.3), not to replace the full Lifshitz validation given separately in Appendix D.

**B.1 Dispersion on the Imaginary Frequency Axis**

The dispersive magnetodielectric response on the imaginary frequency axis is modeled by the Drude–Lorentz forms

$$\begin{matrix} & \varepsilon(i\xi)=1+\frac{\omega_{pe}^{\text{ }2}}{\xi(\xi+\gamma_{e})}, & & \text{(B1)} \end{matrix}$$

$$\begin{matrix} & \mu(i\xi)=1+\frac{F_{m}\omega_{pm}^{\text{ }2}}{\omega_{0m}^{\text{ }2}+\xi(\xi+\gamma_{m})}. & & \text{(B2)} \end{matrix}$$

In the numerical illustrations used here, the parameters are chosen as

$$\begin{matrix} & \omega_{pe}=1.37\times{10}^{16}\text{ }s^{-1},\gamma_{e}=5.0\times{10}^{13}\text{ }s^{-1}, & & \text{(B3)} \end{matrix}$$

$$\begin{matrix} & \omega_{pm}=5.00\times{10}^{15}\text{ }s^{-1},\omega_{0m}=3.00\times{10}^{15}\text{ }s^{-1},\gamma_{m}=5.0\times{10}^{13}\text{ }s^{-1},F_{m}=0.6. & & \text{(B4)} \end{matrix}$$

These functions provide a simple but physically meaningful dispersive model for a passive magnetodielectric cavity. In particular, $\varepsilon(i\xi)$dominates at low frequencies and both $\varepsilon(i\xi)$and $\mu(i\xi)$approach unity at sufficiently large $\xi$, as required for passive behavior on the imaginary axis.

**Figure B1.** Imaginary-axis dispersion of the Drude–Lorentz magnetodielectric cavity used in the revised QTTO illustrations. The dielectric response dominates at low frequencies, while both $\varepsilon(i\xi)$and $\mu(i\xi)$approach unity at large $\xi$, consistent with passive dispersive behavior.

**B.2 Corrected Zero-Temperature Lifshitz Pressure**

The zero-temperature Casimir pressure between two identical planar dispersive half-spaces is used here as the reference pressure

$$\begin{matrix} & P_{0}(d)\equiv P(d,0). & & \text{(B5)} \end{matrix}$$

In the revised numerical treatment, this quantity is computed from the corrected zero-temperature Lifshitz integral using proper numerical integration over the imaginary-frequency axis. This correction is important because the same $P_{0}$ is used to normalize the revised QTTO pressure law and must therefore remain strictly consistent with the finite-temperature Matsubara evaluation used in Appendix D.

Figure B2 shows the magnitude of the corrected zero-temperature pressure as a function of plate separation over the submicron range. The numerical result follows the expected vacuum-dominated trend and provides the physically consistent normalization used throughout the revised manuscript.

**Figure B2.** Corrected zero-temperature Lifshitz pressure $P_{0}(d)$for the Drude–Lorentz magnetodielectric cavity. This reference pressure is used to normalize the revised QTTO law and is computed using proper numerical integration over the nonuniform imaginary-frequency grid.

**B.3 Revised QTTO Temperature Dependence under Geometric Scaling**

The revised QTTO pressure law used in the main text is

$$\begin{matrix} & \frac{P^{'}(T)}{P_{0}}=[1-W(\tau)]\rho^{2}+W(\tau)(\frac{T_{\mathrm{eff}}}{T_{\star}})s_{\perp}^{-4}, & & \text{(B6)} \end{matrix}$$

with

$$\begin{matrix} & \tau=\frac{T_{\mathrm{eff}}}{T_{\star}},W(\tau)=1-\exp(-\tau^{\nu}). & & \text{(B7)} \end{matrix}$$

Here,

$$\begin{matrix} & \rho=\frac{s_{z}}{s_{\perp}} & & \text{(B8)} \end{matrix}$$

is the longitudinal-to-transverse geometric compression ratio, $T_{\mathrm{eff}}$is the metric-induced effective statistical temperature, and $T_{\star}$is the reference crossover scale of the cavity. As emphasized in the main text and Appendices A and C, $T_{\star}$is not a second effective temperature; it is the characteristic scale governing the onset of thermal dominance in the normalized pressure law.

To illustrate how the revised QTTO pressure depends on temperature and geometry, Figure B3 presents $\mid P^{'}(T)\mid$for representative temperatures

$$T=300, 3000, 10000K$$

and longitudinal compression ratios

$$\rho=0.8, 1.0, 1.2$$

with

$$s_{\perp}=1$$

held fixed. In these examples, the temperature dependence enters explicitly through the classical branch $(T_{\mathrm{eff}}/T_{\star})s_{\perp}^{-4}$, while the zero-temperature branch remains controlled by $\rho^{2}$.

The figure highlights three features of the revised law. First, at sufficiently low temperature, the pressure is dominated by the quantum branch and approaches $\rho^{2}$. Second, as temperature increases, the crossover function $W(\tau)$ gradually transfers weight to the thermally populated branch. Third, in the high-temperature regime, the pressure grows approximately linearly with $T_{\mathrm{eff}}/T_{\star}$, modulated by the geometric factors entering the classical sector.

Thus, the revised QTTO law makes explicit that geometry and thermal population play distinct but coupled roles: $\rho$controls the vacuum branch, while $s_{\perp}$ and $T_{\mathrm{eff}}$ jointly control the thermally dominated branch.

**Figure B3.** Revised QTTO-scaled pressure $\mid P^{'}(T)\mid$as a function of separation $d$for representative temperatures $T=300, 3000, 10000K$ and longitudinal compression ratios $\rho=0.8, 1.0, 1.2$, with $s_{\perp}=1$ fixed. The curves are computed from the revised QTTO pressure law using the corrected zero-temperature Lifshitz pressure $P_{0}(d)$as the normalization reference.

**B.4 Parameter-Space Structure of the Revised QTTO Law**

To further visualize the role of geometric anisotropy, the revised QTTO pressure may be plotted over the $(\rho,s_{\perp})$ parameter space at a fixed value of the crossover variable $\tau=T_{\mathrm{eff}}/T_{\star}$. In this representation, the pressure surface reflects the competition between enhancement of the vacuum branch through $\rho^{2}$ and modulation of the thermal branch through $s_{\perp}^{-4}$.

At fixed $\tau$, the revised QTTO law may be viewed as a weighted geometric surface,

$$\begin{matrix} & \frac{P^{'}}{P_{0}}=[1-W(\tau)]\rho^{2}+W(\tau)\text{ }\tau\text{ }s_{\perp}^{-4}, & & \text{(B9)} \end{matrix}$$

which combines these two trends in a single reduced thermodynamic representation.

Figure B4 illustrates this surface at the representative crossover value

$$\begin{matrix} & \tau=\frac{T_{\mathrm{eff}}}{T_{\star}}=1. & & \text{(B10)} \end{matrix}$$

The resulting topology shows how the two geometric scales act differently in the revised model: longitudinal compression strengthens the vacuum-like sector, whereas transverse contraction strengthens the thermally weighted sector.

**Figure B4.** Revised QTTO pressure surface $P^{'}(T)/P_{0}$over the $(\rho,s_{\perp})$parameter space at fixed crossover variable $\tau=T_{\mathrm{eff}}/T_{\star}=1$. The surface illustrates the combined action of the geometric vacuum branch $\rho^{2}$and the thermally weighted classical branch $\tau s_{\perp}^{-4}$.

**Summary of Appendix B**

This appendix illustrates the numerical behavior of the revised QTTO pressure law in a Drude–Lorentz magnetodielectric cavity. The material dispersion model on the imaginary-frequency axis remains unchanged, while the pressure normalization is now based on the corrected zero-temperature Lifshitz pressure $P_{0}(d)$. Within this revised framework, the QTTO pressure depends explicitly on the effective statistical temperature through the classical branch and on geometric anisotropy through the factors $\rho$and $s_{\perp}$.

The numerical illustrations in Figs. B3 and B4 are not intended as a replacement for the full Lifshitz validation given in Appendix D. Rather, they provide a compact visualization of how the revised QTTO law organizes the pressure into a vacuum-dominated geometric branch and a thermally populated classical branch, connected by the crossover function $W(\tau)$. In this sense, Appendix B serves as the numerical illustration of the structure of the revised QTTO model itself.

**Appendix C. Statistical-Field Interpretation of the Revised QTTO Pressure Law**

The purpose of this appendix is to provide a statistical-field interpretation of the revised QTTO Casimir pressure law derived in Appendix A and used in Sec. 4 of the main text. While Appendix A presents the operator-based route starting from the thermofield Hamiltonian, the present appendix reformulates the same result in terms of thermally weighted modal populations and the corresponding free-energy structure. In this way, the revised QTTO pressure law is shown not merely as a geometric interpolation, but as the reduced statistical consequence of combining a vacuum-dominated branch with a thermally populated branch in the transformed optical metric.

**C.1 From the Thermofield Hamiltonian to a Statistical Ensemble**

The QTTO framework extends conventional transformation optics from a purely geometric mapping to a statistical field theory in curved optical space. Instead of treating the transformation only as a reshaping of coordinates in Maxwell’s equations, QTTO assigns to each transformed optical metric a thermofield ensemble in which the modal occupation statistics depend explicitly on the metric-induced effective temperature. This point is already encoded in the main text through the thermofield Hamiltonian and the metric fluctuation–dissipation relation.

The thermodynamic state of the transformed electromagnetic field may therefore be described by an effective density operator of the form

$$\begin{matrix} & \hat{\rho}_{\mathrm{QTTO}}=\frac{\exp[-\hat{H}_{\mathrm{TFD}}^{'}/(k_{B}T_{\mathrm{eff}})]}{\mathrm{Tr}[\exp(-\hat{H}_{\mathrm{TFD}}^{'}/(k_{B}T_{\mathrm{eff}}))]}, & & \text{(C1)} \end{matrix}$$

where $\hat{H}_{\mathrm{TFD}}^{'}$is the transformed thermofield Hamiltonian and $T_{\mathrm{eff}}$is the effective statistical temperature introduced in Eq. (2.6) of the main text.

This expression should not be interpreted as stating that the external bath temperature itself is changed by geometry. Rather, $T_{\mathrm{eff}}$is the temperature parameter that governs the transformed field correlations and modal populations after the coordinate mapping. In this sense, the statistical structure of QTTO is inherited directly from the transformed thermofield correlators.

The corresponding reduced free-energy functional may be written schematically as

$$\begin{matrix} & \mathcal{F}_{\mathrm{QTTO}}(T)=-k_{B}T_{\mathrm{eff}}\ln\mathrm{Tr}[\exp(-\hat{H}_{\mathrm{TFD}}^{'}/(k_{B}T_{\mathrm{eff}}))]. & & \text{(C2)} \end{matrix}$$

The Casimir pressure is then obtained as the derivative of the free energy with respect to the plate separation,

$$\begin{matrix} & P^{'}(T)=-\frac{\partial\mathcal{F}_{\mathrm{QTTO}}}{\partial d}. & & \text{(C3)} \end{matrix}$$

At this level, the pressure is already seen to be both geometric and statistical: geometry enters through the transformed Hamiltonian and modal spectrum, while thermal occupation enters through $T_{\mathrm{eff}}$.

**C.2 Two Statistical Branches of the Pressure**

Within this thermofield ensemble, the pressure naturally decomposes into two asymptotic sectors.

The first is the zero-point sector, which dominates when the modal population is vacuum-like. In this branch, the pressure is controlled by the transformed vacuum spectrum and reduces to the geometric scaling law

$$\begin{matrix} & \frac{P_{q}^{'}}{P_{0}}=\rho^{2},\rho=\frac{s_{z}}{s_{\perp}}. & & \text{(C4)} \end{matrix}$$

The second is the thermally populated sector, which dominates when the transformed modal occupation follows the Rayleigh–Jeans limit,

$$\begin{matrix} & n_{B}(\omega^{'},T_{\mathrm{eff}})\approx\frac{k_{B}T_{\mathrm{eff}}}{\hbar\omega^{'}}. & & \text{(C5)} \end{matrix}$$

Because the thermal population is explicitly proportional to $T_{\mathrm{eff}}$, the corresponding pressure branch must also remain explicitly proportional to $T_{\mathrm{eff}}$. The geometric dependence of this branch is governed primarily by the transverse scale factor, yielding

$$\begin{matrix} & \frac{P_{\mathrm{cl}}^{'}(T)}{P_{0}}=(\frac{T_{\mathrm{eff}}}{T_{\star}})s_{\perp}^{-4}, & & \text{(C6)} \end{matrix}$$

where $T_{\star}$is the reference crossover scale of the dispersive cavity introduced in the main text and Appendix A.

Thus, the statistical-field interpretation leads to the same two-branch structure already identified operatorially:

- a vacuum branch governed by geometric compression,
- and a classical branch governed by thermal population weighted by the effective temperature and modulated by transverse scaling.

**C.3 Statistical Meaning of the Weighting Function**

To combine these two branches in reduced analytical form, we introduce the thermal weighting function $W(\tau)$, where

$$\begin{matrix} & \tau=\frac{T_{\mathrm{eff}}}{T_{\star}}. & & \text{(C7)} \end{matrix}$$

The role of $W(\tau)$is to represent the fractional contribution of the thermally populated sector to the total pressure. At the reduced statistical level, it may be interpreted as

$$\begin{matrix} & W(\tau)=\frac{\int_{0}^{\infty} d\omega\text{ }n_{B}(\omega,T_{\mathrm{eff}})\text{ }D(\omega)}{\int_{0}^{\infty} d\omega\text{ }[\frac{1}{2}+n_{B}(\omega,T_{\mathrm{eff}})]D(\omega)}, & & \text{(C8)} \end{matrix}$$

where $D(\omega)$denotes the transformed modal density relevant to the Casimir stress.

In the present QTTO reduction, the full spectral integral is represented by the smooth crossover form

$$\begin{matrix} & W(\tau)=1-\exp(-\tau^{\nu}), & & \text{(C9)} \end{matrix}$$

with $\nu$controlling the sharpness of the transition between the vacuum-dominated and thermally populated regimes.

**Figure C1.** Statistical interpretation of the revised thermal weighting function $W(\tau)=1-\exp(-\tau^{\nu})$, plotted versus the crossover variable $\tau=T_{\mathrm{eff}}/T_{\star}$. The shaded regions indicate the vacuum-dominated regime $(\tau<1)$and the thermally dominated regime $(\tau>1)$. In the revised QTTO framework, $W(\tau)$represents the gradual redistribution of pressure weight from the zero-point sector to the thermally populated sector.

Figure C1 visualizes the statistical meaning of the crossover function. For $\tau<1$, the vacuum-like sector dominates and the thermally populated contribution remains weak. For $\tau>1$, the thermal sector acquires increasing statistical weight and progressively governs the pressure response. In this sense, $W(\tau)$is not an exact substitute for the full Matsubara-frequency structure at every temperature, but a reduced statistical representation of how pressure weight is transferred from the zero-point sector to the thermal sector as the transformed field population increases.

**C.4 Revised QTTO Pressure Law from Statistical Weighting**

With the two branches identified and the thermal weighting function defined, the total pressure is written as the weighted sum

$$\begin{matrix} & P^{'}(T)=[1-W(\tau)]P_{q}^{'}+W(\tau)P_{\mathrm{cl}}^{'}(T). & & \text{(C10)} \end{matrix}$$

Substituting Eqs. (C4), (C6), and (C9) yields

$$\begin{matrix} & \frac{P^{'}(T)}{P_{0}}=[1-W(\tau)]\rho^{2}+W(\tau)(\frac{T_{\mathrm{eff}}}{T_{\star}})s_{\perp}^{-4},\tau=\frac{T_{\mathrm{eff}}}{T_{\star}},W(\tau)=1-\exp(-\tau^{\nu}). & & \text{(C11)} \end{matrix}$$

This is identical to Eq. (4.3) in the main text and Eq. (A15) in Appendix A. The statistical-field derivation therefore confirms that the revised QTTO pressure law is the reduced ensemble-level form of the same two-sector thermodynamic structure obtained from the operator-based derivation.

**C.5 Interpretation of the Two Limits**

The statistical meaning of the two asymptotic limits is transparent.

For

$$\tau\ll1,$$

the thermal population is negligible, so

$$\begin{matrix} & W(\tau)\to0\Rightarrow\frac{P^{'}(T)}{P_{0}}\to\rho^{2}. & & \text{(C12)} \end{matrix}$$

In this regime, the Casimir pressure is vacuum-dominated and reflects the geometric deformation of the zero-point modal spectrum.

For

$$\tau\gg1,$$

the thermally populated sector dominates, so

$$\begin{matrix} & W(\tau)\to1\Rightarrow\frac{P^{'}(T)}{P_{0}}\to(\frac{T_{\mathrm{eff}}}{T_{\star}})s_{\perp}^{-4}. & & \text{(C13)} \end{matrix}$$

In this regime, the Casimir pressure becomes a thermodynamic stress controlled by thermal modal occupation and geometrically modulated by the transformed optical metric.

Thus, the revised QTTO law is naturally interpreted as the smooth redistribution of statistical weight between a vacuum branch and a classical thermal branch.

**C.6 Role of** $\boldsymbol{T}_{\mathbf{eff}}$**and** $\boldsymbol{T}_{\boldsymbol{\star}}$

It is important to distinguish the physical roles of $T_{\mathrm{eff}}$ and $T_{\star}$.

The effective temperature $T_{\mathrm{eff}}$is the transformed statistical temperature that enters the thermofield correlators, modal occupation numbers, and fluctuation–dissipation structure. It is therefore the temperature controlling the actual population of the mapped modes.

The scale $T_{\star}$, by contrast, is not a second temperature of the field. It is a reference crossover scale of the cavity, introduced because the finite-temperature branch is normalized by the zero-temperature reference pressure $P_{0}$. Physically, it characterizes the temperature range over which thermal occupation becomes sufficiently important to compete with the vacuum branch in the pressure law.

This distinction is essential. The pressure depends on $T_{\mathrm{eff}}$because the field correlations depend on $T_{\mathrm{eff}}$. The crossover variable $\tau=T_{\mathrm{eff}}/T_{\star}$ is introduced only to encode when the thermal sector becomes dominant relative to the vacuum sector.

**Summary of Appendix C**

Appendix C provides the statistical-field interpretation of the revised QTTO pressure law. Starting from a thermofield ensemble in the transformed optical metric, the pressure is understood as the weighted combination of two asymptotic statistical sectors: a geometric vacuum branch and a thermally populated classical branch. The vacuum branch scales as

$$\frac{P_{q}^{'}}{P_{0}}=\rho^{2},$$

while the classical branch scales as

$$\frac{P_{\mathrm{cl}}^{'}(T)}{P_{0}}=(\frac{T_{\mathrm{eff}}}{T_{\star}})s_{\perp}^{-4}.$$

A smooth thermal weighting function $W(\tau)$, with $\tau=T_{\mathrm{eff}}/T_{\star}$, redistributes the pressure contribution between these two sectors and yields the revised law

$$\frac{P^{'}(T)}{P_{0}}=[1-W(\tau)]\rho^{2}+W(\tau)(\frac{T_{\mathrm{eff}}}{T_{\star}})s_{\perp}^{-4}.$$

The revised QTTO pressure law should therefore be understood as a reduced statistical representation of the pressure crossover between vacuum-dominated and thermally dominated regimes in a transformed optical metric.

**Appendix D. Numerical Validation Against Finite-Temperature Lifshitz Theory**

This appendix presents the numerical validation of the revised QTTO pressure law against the finite-temperature Lifshitz–Matsubara formulation for a dispersive Drude–Lorentz magnetodielectric cavity. The purpose of this comparison is not to claim an exact point-by-point replacement of the full Lifshitz theory, but to test whether the revised QTTO interpolation correctly reproduces the quantum limit, the thermal asymptote, and the overall temperature-driven crossover structure of the Casimir pressure.

**D.1 Numerical Setup**

The comparison is performed for two parallel transformed plates separated by a fixed distance

$$\begin{matrix} & d=200\text{ }nm. & & \text{(D1)} \end{matrix}$$

The dispersive material response on the imaginary frequency axis is modeled by a Drude–Lorentz magnetodielectric form,

$$\begin{matrix} & \varepsilon(i\xi)=1+\frac{\omega_{pe}^{\text{ }2}}{\xi(\xi+\gamma_{e})}, & & \text{(D2)} \end{matrix}$$

$$\begin{matrix} & \mu(i\xi)=1+\frac{F_{m}\omega_{pm}^{\text{ }2}}{\omega_{0m}^{\text{ }2}+\xi(\xi+\gamma_{m})}. & & \text{(D3)} \end{matrix}$$

For the numerical evaluation used here, the parameters are chosen as

$$\begin{matrix} & \omega_{pe}=1.37\times{10}^{16}\text{ }s^{-1},\gamma_{e}=5.0\times{10}^{13}\text{ }s^{-1}, & & \text{(D4)} \end{matrix}$$

$$\begin{matrix} & \omega_{pm}=5.00\times{10}^{15}\text{ }s^{-1},\omega_{0m}=3.00\times{10}^{15}\text{ }s^{-1},\gamma_{m}=5.00\times{10}^{13}\text{ }s^{-1},F_{m}=0.6. & & \text{(D5)} \end{matrix}$$

The zero-temperature reference pressure $P_{0}\equiv P(d,0)$ is computed from the corrected Lifshitz zero-temperature integral, while the finite-temperature Lifshitz pressure $P_{\mathrm{Lif}}(T)$ is evaluated from the corresponding Matsubara summation using the same dispersive model.

**D.2 Corrected Zero-Temperature Normalization**

A crucial point in the numerical comparison is the consistent normalization of the finite-temperature pressure by the zero-temperature reference pressure $P_{0}$. In the revised calculation, the zero-temperature Lifshitz integral is evaluated using proper numerical integration over the nonuniform imaginary-frequency grid. This correction ensures that

$$\begin{matrix} & \frac{P_{\mathrm{Lif}}(T)}{P_{0}}\to1\text{as}T\to0, & & \text{(D6)} \end{matrix}$$

as required for consistency between the zero-temperature and finite-temperature formulations.

After this correction, the Lifshitz pressure satisfies the expected low-temperature limit to high numerical accuracy, thereby providing a reliable benchmark for the revised QTTO comparison.

**D.3 Revised QTTO Comparison**

The revised QTTO pressure law used in the comparison is

$$\begin{matrix} & \frac{P^{'}(T)}{P_{0}}=[1-W(\tau)]\rho^{2}+W(\tau)(\frac{T_{\mathrm{eff}}}{T_{\star}})s_{\perp}^{-4}, & & \text{(D7)} \end{matrix}$$

with

$$\begin{matrix} & \tau=\frac{T_{\mathrm{eff}}}{T_{\star}},W(\tau)=1-\exp(-\tau^{\nu}). & & \text{(D8)} \end{matrix}$$

For the validation shown in this appendix, we consider the identity-mapping case

$$\begin{matrix} & \rho=1,s_{\perp}=1,\mid det\Lambda\mid=1, & & \text{(D9)} \end{matrix}$$

so that

$$\begin{matrix} & T_{\mathrm{eff}}=T. & & \text{(D10)} \end{matrix}$$

The crossover parameters used in the final revised comparison are

$$\begin{matrix} & \nu=2.5,T_{\star}=4.2\times{10}^{3}\text{ }K. & & \text{(D11)} \end{matrix}$$

The value of $T_{\star}$is interpreted here as a **reference crossover scale of the cavity**, not as a second effective temperature. Its role is to encode the onset of thermal dominance in the dispersive cavity after normalization by $P_{0}$. This interpretation is consistent with the revised discussion in Sec. 4 and Appendix A.

**D.4 Comparison with Lifshitz Theory**

Figure D1 compares the normalized revised QTTO pressure $P^{'}(T)/P_{0}$ with the normalized finite-temperature Lifshitz pressure $P_{\mathrm{Lif}}(T)/P_{0}$ over the temperature range from ${10}^{-1}\text{ }K$to ${10}^{4}\text{ }K$.

The comparison shows three important features:

1. **Low-temperature agreement.**

In the quantum regime, both QTTO and Lifshitz approach the same normalized vacuum limit,

$$\begin{matrix} & \frac{P(T)}{P_{0}}\approx1,T\ll T_{\star}. & & \text{(D12)} \end{matrix}$$

2. **High-temperature agreement.**

In the thermally dominated regime, the revised QTTO law reproduces the growth of the Lifshitz pressure and accurately captures the large-$T$ asymptotic trend,

$$\begin{matrix} & \frac{P(T)}{P_{0}}\sim\frac{T}{T_{\star}},T\gg T_{\star}. & & \text{(D13)} \end{matrix}$$

3. **Intermediate crossover behavior.**

Moderate deviations remain in the crossover region, where the full Matsubara summation retains spectral details that are not resolved explicitly by the reduced QTTO interpolation. These deviations are expected, because the revised QTTO law is designed as a compact thermodynamic crossover model rather than an exact pointwise reconstruction of the full Lifshitz spectrum.

Thus, the numerical comparison confirms that the revised QTTO law correctly reproduces the two asymptotic regimes and provides a compact analytical description of the overall thermal crossover.

**Figure D1.** Comparison between the revised QTTO normalized pressure law and the finite-temperature Lifshitz result at fixed separation $d=200\text{ }\mathrm{nm}$. Both curves are normalized by the same corrected zero-temperature reference pressure $P_{0}$. The QTTO curve is obtained from the revised thermodynamic interpolation law of Eq. (D7), with $T_{\mathrm{eff}}=T$, $\nu=2.5$, and $T_{\star}=4.2\times{10}^{3}\text{ }K$, while the Lifshitz curve is computed from the full Matsubara summation using the same Drude–Lorentz dispersive model. The comparison shows excellent agreement in the low- and high-temperature limits and captures the overall temperature-driven crossover in compact analytical form.

**D.5 Interpretation of the Validation**

The significance of Figure D1 is not that the revised QTTO law replaces the full Lifshitz theory at every temperature point, but that it preserves the physically correct structure of the crossover between the vacuum-dominated and thermally dominated regimes. In particular:

- the zero-temperature branch remains anchored to the correct Lifshitz normalization,
- the classical branch restores the explicit linear dependence on $T_{\mathrm{eff}}$,
- and the crossover scale is controlled by the cavity reference temperature $T_{\star}$, rather than being tied directly to a bare plasma-frequency scale.

This confirms that the revised QTTO formulation should be interpreted as a **self-consistent reduced thermodynamic model** of the finite-temperature Casimir pressure in transformed optical media.

**Summary of Appendix D**

Appendix D validates the revised QTTO pressure law against the full finite-temperature Lifshitz–Matsubara theory for a dispersive Drude–Lorentz cavity. Using a corrected zero-temperature normalization and a cavity-based crossover scale $T_{\star}$, the revised QTTO law reproduces the correct vacuum limit, captures the thermal asymptote, and provides a compact analytical representation of the temperature-driven crossover of the Casimir pressure. The remaining deviations in the intermediate regime reflect the reduced character of the QTTO interpolation relative to the full spectral detail retained by the Lifshitz summation.
